# JUND-driven stress-responsive astrocytes promote neuronal apoptosis via enhanced gap junction signaling in autism spectrum disorder

**DOI:** 10.1186/s13229-026-00717-0

**Published:** 2026-05-19

**Authors:** Qi Wang, Huamin Yin, Qi Jiang, Yubo Qi, Jieli Bai, Zhendong Wang, Kailai Liu, Ruizhen Sun, Wenhang Wang, Canying Liu, Weishuo Yan, Jin Luo, Lian Duan, Zhiyan Shan

**Affiliations:** 1 Department of Histology and Embryology, School of Basic Medical Sciences, Harbin Medical University, Harbin, China; 2 Central Laboratory, The First Affiliated Hospital of Wenzhou Medical University, Wenzhou, China; 3 Guangzhou National Laboratory, Guangzhou, China

**Keywords:** Autism spectrum disorder, Astrocyte, Stress, JUND, Gap, Apoptosis

## Abstract

**Background:**

Dysregulated stress responses are increasingly implicated in the pathophysiology of autism spectrum disorder (ASD). Astrocytes, which are highly vulnerable to stress, critically support neuronal survival; however, their specific role in ASD remains poorly defined.

**Methods:**

We analyzed single nucleus RNA sequencing data from postmortem cortices of ASD and control donors. Our analysis included enrichment analysis of astrocyte subpopulations and integrated pySCENIC-based GRN reconstruction, TF profiling, regulon-DEG analysis, and cell-cell communication. Mechanistic studies involved human iPSC-derived astrocytes-neurons co-culture and astrocyte-specific *JUND* overexpression in mice. Functional and behavioral assessments were performed to evaluate neuronal viability and ASD-relevant phenotypes.

**Results:**

A subpopulation of stress-responsive astrocytes (SRAs) was identified as specifically enriched in ASD, displaying early transcriptional activation and increased abundance. These SRAs showed marked upregulation of both stress-response pathways and distinct reactive signatures. JUND was established as the core transcriptional orchestrator, controlling 23.8% of the dysregulated transcriptome that defines the core stress-responsive signature, including multiple ASD risk genes. *JUND* overexpression in astrocytes recapitulated the SRAs molecular profile, while astrocyte-specific JUND activation in mouse cortex elicited core ASD-like behaviors. Mechanistically, JUND upregulated the gap junction gene *GJA1*, enhancing astrocyte-neuron communication and promoting neuronal apoptosis. This pathogenic cascade was rescued by the gap junction inhibitor GAP27.

**Limitations:**

This study is focused solely on specific brain regions, the generalizability of the JUND-GJA1-GAP axis across diverse ASD subtypes remains to be validated, and the complete activation pathway of JUND signaling within SRAs as well as their interaction mechanisms with other glial cells are not well understood.

**Conclusions:**

Our findings demonstrate that a specialized astrocyte subpopulation mediates stress-induced dysfunction in ASD via a JUND-GJA1-GAP apoptotic signaling axis, providing a translational anchor for targeting astrocyte-specific pathways in ASD therapy.

**Supplementary Information:**

The online version contains supplementary material available at 10.1186/s13229-026-00717-0.

## Introduction

Autism spectrum disorder (ASD) is a neurodevelopmental condition whose rising global prevalence underscores an urgent public health challenge [1, 2]. While core behavioral manifestations, social communication deficits and restricted repetitive behaviors, are well-established, the underlying neuropathophysiology remains poorly defined [3–5]. Notably, heightened sensitivity to benign environmental stimuli in a subset of individuals points to dysregulated stress response systems as a potential convergent mechanism [6]. However, traditional research on stress neurobiology has heavily focused on neurons but overlooked astrocytes, which are critical for synaptic regulation and neuronal metabolic support [7, 8].

Nevertheless, compelling evidence shows that when exposed to stress-inducing stimuli, astrocytes can sense this stress, interact with neurons, and actively regulate their responses [9–11]. Human neuroimaging and postmortem studies indicate that stress induces brain region-specific alterations in astrocyte density and morphology [12, 13]. First, glial cell density varies across psychiatric conditions. For instance, the reduction in glial cell density correlates with major depressive disorder (MDD) [14], whereas no significant change is observed in bipolar disorder (BD) [15]. Second, studies have documented specific structural modifications in astrocytes in response to stress. Acute stress leads to an increase in astrocyte volume, whereas chronic unpredictable mild stress results in astrocytic atrophy [16]. Notably, chemical ablation of glial cells in the prefrontal cortex elicits depression-like behaviors that mirror those induced by chronic stress [17]. These findings highlight the involvement of astrocytes in stress-induced maladaptive neural plasticity and behavioral abnormalities. Furthermore, transcriptomic studies reveal substantial overlap between ASD-risk genes and astrocytic markers dysregulated in stress-related disorders [18, 19], suggesting a plausible link between astrocyte stress responses and ASD etiology.

At the molecular level, cellular stress adaptation is orchestrated by specialized transcription factors (TFs) that reprogram gene expression in pathology-specific manners [20, 21]. Although such TFs represent promising mechanistic hubs, those directing astrocyte stress responses in ASD remain unknown. Adding further complexity, astrocytes display profound functional and transcriptional heterogeneity across and within brain regions [22–24]. This diversity enables specialized astrocyte subpopulations to fulfill distinct roles within neuro-glial circuits [25], as illustrated by the recent identification of a subpopulation specifically involved in modulating glutamatergic transmission [26]. Importantly, the phenomenon where distinct astrocyte subpopulations underpin specific disease processes is well-established in other neuropsychiatric disorders, including Huntington’s disease (HD) and Alzheimer’s disease (AD) [27–29]; however, this area remains minimally investigated in ASD. Existing studies have primarily focused on mapping the spatial distribution of astrocyte subpopulations in the brain and their reactive inflammatory responses in ASD, which was insufficient to elucidate the role of astrocyte heterogeneity in the complex pathological processes underlying ASD [30, 31].

Here, through integrated single-nucleus transcriptomic and functional analyses, we identify a previously unrecognized subpopulation of stress-responsive astrocytes (SRAs) enriched in ASD and governed by the transcription factor JUND. We demonstrate that JUND activation drives aberrant GJA1-mediated gap junction formation between SRAs and excitatory neurons, resulting in neuronal apoptosis via coordinated induction of BAX/caspase3 and suppression of BCL2/BCL-xL. Crucially, astrocyte-specific *JUND* overexpression in the mouse prefrontal cortex (PFC) recapitulates core ASD-like behavioral phenotypes. These findings not only elucidate a novel astrocyte-mediated pathway contributing to ASD pathogenesis but also highlight JUND-driven SRAs as a potential therapeutic target for modulating stress-related neural dysfunction in ASD.

## Materials and methods

### Acquisition and preprocessing of snRNA-seq data

The single-nucleus RNA sequencing (snRNA-seq) data used in this study are publicly available through the UCSC Cell Browser (https://cells.ucsc.edu/?ds=autism) [32]. A total of 104,559 nuclei from the prefrontal cortex (PFC) and anterior cingulate cortex (ACC) of 22 individuals with ASD and 13 controls were included. Samples were collected from individuals aged 4 to 22 years, with matching for age, gender, and cause of death to ensure group balance. A Wilcoxon rank-sum test (*p* > 0.05) was used to evaluate the consistency of cell type proportions between the two conditions across brain regions.

Based on known cell type markers, the initial eleven neuronal and six non-neuronal subpopulations (original data) were consolidated into six neuronal and five non-neuronal categories. The original neuronal subpopulations, which included parvalbumin (IN-PV), somatostatin (IN-SST), SV2C (IN-SV2C), VIP (IN-VIP) interneurons; layer 2/3 (L2/3) and layer 4 (L4) excitatory neurons; layer 5/6 corticofugal projection (L5/6) and layer 5/6 cortico-cortical projection (L5/6-CC) neurons; maturing neurons (Neu-mat); and two NRGN-expressing subpopulations (Neu-NRGN-I and Neu-NRGN-II), were reclassified as follows: IN-PV, IN-SST, IN-SV2C, and IN-VIP were grouped as interneurons; L2/3 and L4 were merged into excitatory neurons; L5/6 and L5/6-CC were combined as layer 5/6 cortical projection neurons; and Neu-NRGN-I and Neu-NRGN-II were integrated as Neu-NRGN. Among non-neuronal cells, fibrous (AST-FB) and protoplasmic (AST-PP) astrocytes were consolidated into a single astrocyte category. The final non-neuronal classification thus comprises five types: astrocytes, oligodendrocyte precursor cells (OPC), oligodendrocytes, microglia, and endothelial cells.

### Acquisition and preprocessing of transcriptomic data

To assess the enrichment of astrocyte-related transcriptional signatures in ASD, we obtained four publicly available ASD-related transcriptomic datasets from the Gene Expression Omnibus (GEO) database(https://www.ncbi.nlm.nih.gov/geo/) [33–36], which included a total of 53 ASD and 47 control samples from multiple brain regions for transcriptomic analysis (Table S1). The RNA-Seq count data were downloaded directly and converted to TPM values using the hg38 reference genome. All microarray datasets were processed using a standardized computational pipeline. Raw data underwent quality control and normalization using the limma package (v3.62.2) in R. The astrocyte enrichment score for each sample was quantified via ssGSEA, implemented through the GSVA package (v2.0.7). An astrocyte-specific gene set was curated by combining markers identified from the ASD snRNA-seq dataset using the FindMarkers function with previously established molecular markers, including well-characterized astrocyte genes such as *GFAP*, *AQP4*, and *SLC1A3* [37]. Group comparisons between ASD and control samples across datasets were conducted using Wilcoxon rank-sum tests, with multiple testing correction applied via the Benjamini-Hochberg method (false discovery rate (FDR) < 0.05 considered significant).

### Identification of astrocyte subpopulations

To characterize distinct astrocyte subpopulations, astrocyte nuclei were isolated from the integrated dataset based on original cell annotations. All preprocessing steps were performed using the Seurat (v4.3.0) pipeline. Gene expression data were normalized using NormalizeData function with default parameters. Highly variable genes within the astrocyte dataset were identified through variance-stabilizing transformation, and the top 2000 variable genes were selected for dimensionality reduction. Principal component analysis (PCA) was conducted, and the top principal components accounting for significant variance were used as input for Uniform Manifold Approximation and Projection (UMAP). The UMAP embedding was generated using the RunUMAP function with a minimum distance parameter of 0.3. Subsequently, unsupervised clustering was performed on the UMAP coordinates using the Louvain algorithm, with a resolution parameter optimized to 0.65 (selected from a range of 0.4 to 1.2) to delineate potential astrocyte subpopulations. The resultant clusters were visualized in the UMAP space, and marker genes were identified for each cluster to characterize astrocyte subpopulations.

To investigate functional differences among astrocyte subpopulations, we identified DEGs across six clusters by performing pairwise differential expression analysis utilizing the FindAllMarkers function with default parameters. For each cluster, candidate genes (adjusted *p* < 0.05, fold change > 1.5) were subsequently tested for enrichment of statistically significant GO terms using hypergeometric tests. Significance was defined at FDR-corrected *p* < 0.1. Enriched GO terms specific to Cluster1 were emphasized and visualized to elucidate the unique functional characteristics of this subpopulation.

The Monocle3 package was employed to analyze single-cell trajectories for identifying developmental transitions. We embedded the seurat object data information into the monocol3 object and used the learn_graph and order_cells functions for trajectory analysis. Subsequently, visualization was conducted using the plot_cells function. Moreover, we used the VECTOR algorithm (https://raw.githubusercontent.com/jumphone/Vector/master/Vector.R) to validate the inferred trajectories.

### GO and KEGG analysis

We employed the FindMarkers function to identify DEGs between individuals with ASD and controls across all major cell types, including both broad categories and specific astrocyte subpopulations. Subsequently, GSEA was performed on the DEGs from each cell type using the clusterProfiler package (v4.14.6) to identify significantly enriched GO terms and KEGG pathways.

### Astrocyte reactivity analysis

To functionally characterize astrocyte subpopulations, we computed signature scores for pan-reactive, neurotoxic (A1), and neuroprotective (A2) phenotypes using the AddModuleScore function in Seurat. Gene sets for each signature were defined according to previously published studies [38]. The scores were z-normalized across all cells. We then applied Gaussian Mixture Model (GMM) clustering from the mclust R package (v6.0.0) to classify cells into distinct reactive states based on their signature scores. Model selection was performed using the Bayesian Information Criterion (BIC), and the optimal number of components was determined automatically.

### pySCENIC regulatory networks analysis

We utilized pySCENIC (v0.12.0, https://pyscenic.readthedocs.io) to analyze astrocyte subpopulations. The Seurat object was converted into an AnnData object and used as an input to the pyScenic pipeline. The expression counts matrix was used as input and processed with default parameters. Gene Regulatory Networks (GRNs) were constructed using the GRNBoost2 algorithm with a comprehensive transcription factor database (RCIStarget). These GRNs were refined to include only downstream targets with a TF-binding motif located within 10 kb of the Transcription Start Site (TSS). Regulon prediction was conducted using cisTarget motif annotations and hg38.feather ranking databases. Area Under the Curve (AUC) scores were then calculated to quantify the activity of each gene signature across single cells using the default threshold, and Regulon specificity scores (RSS) of each astrocyte subpopulation were determined using the regulon_specificity_scores function. For marker genes in each astrocyte subpopulation, the top five genes in ASD and control are presented. Finally, all pyScenic results were integrated into an AnnData object.

To assess the overlap of regulatory elements influencing transcriptional changes in astrocyte Cluster1, we compared the top 5 significantly differentially expressed TFs in ASD astrocyte Cluster1 with the upregulated DEGs in ASD. We then identified the top 4 most enriched regulatory elements in the DEGs of ASD astrocyte Cluster1 to determine the proportion of these elements accounting for the changes observed in the overall DEGs.

### JUND regulon analysis

Protein-protein interaction (PPI) analysis was performed on the upregulated DEGs in ASD putatively regulated by JUND, using the STRING database (v12.0), with a minimum required interaction score set to 0.70 (high confidence). The resulting network was visualized in Cytoscape (v3.9.1). Subsequently, GO enrichment analysis (covering Biological Process, Molecular Function, and Cellular Component domains) and KEGG pathway enrichment analysis were conducted for these DEGs using the clusterProfiler R package. Terms with an adjusted *p* < 0.05 following Benjamini-Hochberg correction were considered statistically significant.

### Cell-cell communication analysis

We employed CellChat to systematically analyze cell-cell communication between astrocyte Cluster1 and excitatory neuronal subpopulations. For each cell type pair, we identified and quantified ligand-receptor (L-R) interactions, including multimeric complexes. Cellular communication was inferred based on predicted ligand and receptor expression patterns, where the expression levels were approximated by the geometric mean within each cell type. These interactions represent the communication probability (as defined in CellChat) between all expressed ligands in one cell type and their corresponding receptors in another. Notably, CellChat incorporates key signaling components such as heteromeric complexes beyond simple L-R pairs; thus, the absence of any essential subunit results in no inferred interaction. The genes and interaction used for subsequent analysis were retained by subsetData, identifyOverExpressedGenes, and identifyOverExpressedInteractions functions with default paramters.

The overall information flow of a signaling pathway was defined as the sum of the communication probabilities of all ligand-receptor pairs within that pathway. To compare the intergroup differences in cell communicaion, we merged the CellChat objects by liftCellChat and mergeCellChat function.

The signaling roles of each cell population (e.g., sender, receiver, mediator, and influencer) within specific pathways such as the Gap Junction pathway were systematically identified based on the inferred communication networks. The relative contribution of each ligand-receptor pair to a given signaling pathway was quantified as the ratio of its communication probability to the total communication probability of all pairs within that pathway. To evaluate the differential signaling behavior of astrocyte Cluster1 as a sender toward various excitatory neuronal subpopulations between ASD and control groups, we compared the interaction strengths of all ligand-receptor pairs originating from astrocyte Cluster1 and targeting excitatory neurons by identifyOverExpressedGenes and netMappingDEG functions with default paramters. The results were visualized using netVisual_bubble function to illustrate the altered signaling patterns.

### Mice

Adult male wild-type C57BL/6N mice (5 weeks old, 18–20 g) were purchased from Beijing Weitong Lihua Experimental Animal Technology Co., Ltd. All mice were maintained in a temperature-controlled environment with a temperature of 22–25℃, humidity of 50%, and a 12-hour light/dark cycle.

### Stereotaxic injection for astrocyte-specific plasmid delivery

Gene delivery to mouse cortical astrocytes was performed using a previous metabolite-mediated protocol [39]. Briefly, mice were anesthetized with pentobarbital (100 mg/kg, i.p.) and secured in a stereotactic frame with body temperature maintained at 37 °C. The eyes were protected with chlortetracycline hydrochloride ointment, and the skull was disinfected with 3% hydrogen peroxide. Using the bregma and lambda as references, the head was leveled horizontally. A 1.2 µL gDAM mixture of plasmid (1 µg/µL; PLV3-CMV-mCherry-*JUND*-Puro or PLV3-CMV-mCherry-Puro; MIAOLING) and 300 mM glycine (solarbio) was then injected into the medial prefrontal cortex (mPFC) (AP: +1.94 mm, ML: ±0.35 mm, DV: − 2.5 mm) at 1 nL/s [40].

### Mouse behavioral tests

Mice underwent behavioral assessment 24 h before and 6 days after stereotactic brain injection.

#### Three-chamber social test

Sociability and response to social novelty test was performed as previously described with minor modifications [41]. The three-chamber apparatus consisted of three adjacent compartments, each measuring 40 × 60 × 22 cm. During the habituation phase, each mouse was allowed to freely explore the empty apparatus for 10 min. Next, the mouse was gently confined in the center chamber while an inverted metal cup (object, O) and another chamber where a wild-type stranger mouse (stranger 1, S1) held was placed in left and right chamber, respectively. The test mouse was then allowed to explore both chambers for 10 min Preference for social novelty was assayed in a third 10 min period by introducing a novel mouse (stranger 2, S2) into left chamber. The behavior of each mouse was recorded and analyzed using Noldus EthoVision XT software. The total time spent in each chamber was quantified to evaluate social preference and social novelty. Sociability index=Time (S1 − O) / (S1 + O), social novelty index=Time (S2 − S1) / (S2 + S1).

#### Marble burying test and self-grooming behavior

Test cages were filled with 5 cm deep wood chip bedding and 20 glass marbles were placed on the bedding surface, evenly spaced. All mice were placed in the same corner of the cage and allowed to explore freely. After 30 min, the mice were removed from the cage and each cage was photographed for subsequent analysis. The number of marbles buried (to 2/3 their depth) was counted.

Self-grooming behavior was defined as stroking or scratching of the body or face, or licking body parts. Mice were individually placed in the cage and grooming behavior was recorded for 10 min.

### NPCs differentiation and culture

Human induced pluripotent stem cell (iPSC) lines (DYR0100) were maintained in mTeSR1 medium (STEMCELL Technologies) on Matrigel-coated plates. Neural progenitor cells (NPCs) differentiation was performed using the STEMdiff™ SMADi Neural Induction Kit (STEMCELL Technologies). Briefly, 2–3 × 10^6^ iPSCs were suspended in neural induction medium and seeded into AggreWell 800 plates (STEMCELL Technologies) for embryoid body (EB) formation. Medium was relaced daily for 5 days. On day 5, EBs were collected and replated onto fresh Matrigel-coated plates for 7 days to facilitate neural rosette (NR) formation. NRs were selectively isolated using the STEMdiff™ Neural Rosette Selection Reagent (STEMCELL Technologies), and the resulting cells were further cultured in neural induction medium for 4–6 days to establish NPCs. NPCs were maintained and expanded in STEMdiff™ Neural Progenitor Medium (NPM).

### Generation of astrocyte and neuron

Human NPCs were seeded onto gelatin-coated plates and cultured in astocyte medium (ScienCell), with medium refreshed every two days. After ten days of differentiation, cells were analyzed by flow cytometry for CD184 and CD44 expression. CD184 + CD44+ astrocyte were isolated and expanded through 2–3 passages to achieve high purity. For neuron induction, NPCs were seeded onto poly-ornithine/laminin (Sigma) -coated plates and cultured in NPM. To enable subsequent neuronal isolation in neuron-glia co-cultures, a defined microtopography was created on the well surface prior to matrix coating. Two parallel grooves were mechanically etched into the well bottom using a syringe needle under controlled pressure, with the displaced plastic forming raised flanking supports. After 1 day, these cells were cultured in DMEM/F-12 medium plus N2 supplement (Gibco), B27 supplement (Gibco), 20 ng/mL BDNF (Peprotech), 20 ng/mL GDNF (Gibco) and 1 mM cAMP (Sigma). The medium was changed every 2 days. For co-culture experiments, were performed on day10 of differentiation. On day 10 post-induction, glial cells were co-cultured with neurons.

### Lentiviral production

Lentiviral constructs for control (PLV3-CMV-mCherry-Puro) and OE-*JUND* (PLV3-CMV-mCherry-JUND-Puro) were purchased from MIAOLING BIOLOGY. To obtain recombinant lentiviruses, 293T cells were transfected using Lipo8000™ Transfection Reagent (Beyotime Biotechnology) with three plasmids: two helper plasmids (pCMV-VSVG and psPAX2) and the respective transfer plasmid. The transfection procedure was performed according to the manufacturer’s instructions. The lentiviral supernatant was harvested 48 h post-transfection and filtered through a 0.45 μm membrane.

### Astrocyte infection and neuron-astrocyte co-culture

Human iPSCs-derived astrocytes were plated onto gelatin-coated coverslips. The next day, upon reaching approximately 80% confluence, the cells were infected with a 1:1 mixture of fresh culture medium and lentiviral stock. After second infection, the coverslips with adhering astrocytes were flipped and placed face-down onto the human iPSCs-derived neuron culture dishes to establish a direct contact co-culture system, ensuring that the astrocytes and neurons were facing each other without separation by the glass. On the second day of co-culture, the medium was replaced with fresh neuronal culture medium, with or without the connexin43 mimetic peptide GAP27 (500 µM, MCE). The co-culture was then maintained for an additional 24 h prior to neuronal harvesting and analysis.

### Immunofluorescence

Samples were fixed in 4% PFA for 30 min at RT and permeabilized with 0.25% Triton X-100 for 15 min. After blocking with 3% blocking serum for 1 h, samples were incubated with primary antibodies at 4℃ overnight. The samples were then stained with fluorophore-conjugated secondary antibodies for 1 h at RT. Nuclei were dyed counterstained with Hoechst33342 (Beyotime Biotechnology), and images were acquired using a fluorescence microscope (Nikon). Detailed antibody information is provided in Table S2.

### Western blot analysis

Western blot was performed according to standard protocols. Briefly, proteins were extracted from neurons co-cultured with astrocyte. Following determination of protein content, aliquots were mixed with an equal volume of loading buffer. Total protein are resolved by 12% SDS-PAGE and electroblotted onto PVDF membranes (Millipore). The membranes were incubated with primary antibody overnight at 4℃. Elution, secondary antibody incubated for 1 h at RT. Aimed protein bands were visualized through ECL (Thermo Fisher) and quantified using ImageJ software. Additional antibody information is available in Table S2.

### Quantitative real-time PCR

Total RNA was extracted from cells using Trizol reagent (Invitrogen). Reverse transcription was performed using the One-Step gDNA Removal (TransGen). Quantitative real-time PCR (qPCR) was performed using SYBR supermix (TransGen) following the manufacturer’s instructions. The primers used in qPCR analysis were listed in Table S3.

### Cell-apoptosis detection

Apoptosis was analyzed using a commercial apoptosis detection kit (Beyotime Biotechnology) according to the manufacturer’s protocol. The percentage of apoptotic neurons was quantified by counting Annexin V-positive cells under a fluorescence microscope.

### Experimental design and statistical analysis

All experiments included at least three biological replicates per group. Data distribution was assessed for normality using the Kolmogorov-Smirnov test. Group comparisons were performed with unpaired two-tailed Student’s t-tests (for two groups) or one-way ANOVA (for three or more groups) using GraphPad Prism 6.0. Data are expressed as mean ± SEM, with significance defined as *p < 0.05, **p < 0.01, ***p < 0.001, and ****p < 0.0001.

## Result

### Astrocytic enrichment and stress response signatures in the ASD cortex

**Fig. 1:**
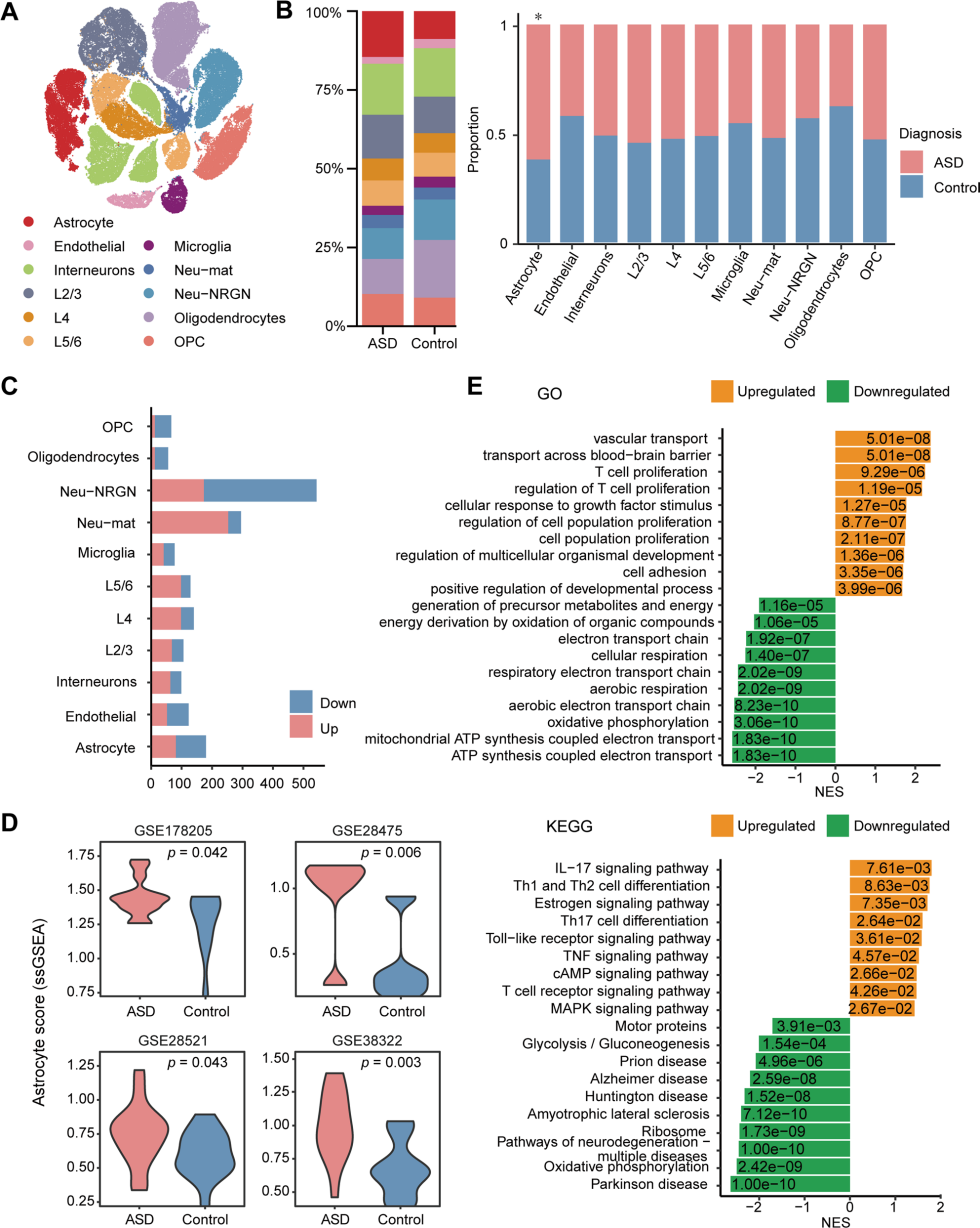
snRNA-seq data show increased abundance of astrocytes in ASD cortex.** (A)** Unbiased clustering of snRNA-seq data and cell type annotation based on established marker genes. **(B)** A grouped stacked bar chart (left) and a proportional stacked bar chart (right) show the proportions of eleven major cell types in ASD and controls. *p < 0.05 **(C)** Bar plots illustrate the number of DEGs across the eleven cell types (FDR < 0.05, |log_2_FC|>0.25). **(D)** Violin plots represent elevated expression levels of astrocyte markers in ASD brain RNA-Seq datasets. **(E)** GO (top) and KEGG (bottom) enrichment analyses of DEGs in astrocytes

To explore the potential involvement of astrocytes in ASD pathogenesis, we analyzed single-nucleus RNA sequencing (snRNA-seq) data obtained from prefrontal and anterior cingulate cortex tissues, comprising 104,559 cells from 22 individuals with ASD and 13 control subjects from the UCSC database. Using established cellular markers, we identified 11 distinct cell clusters, encompassing six neuronal subpopulations, four glial cell types, and endothelial cells (Fig. 1A). Comparative cellular composition analysis revealed significant astrocyte enrichment in ASD specimens, demonstrating both altered distribution patterns and substantially increased proportional abundance (Fig. 1B; Results S1). Differential expression gene (DEG) analysis further identified 190 significantly dysregulated genes in ASD astrocytes, including key markers such as *GFAP*, *APOE*, and *AQP4* (Fig. 1C, Figure S1). The robustness of these findings was confirmed through replication in four independent RNA-seq data (Fig. 1D; Table S1).

We next asked whether these transcriptional changes reflect functional perturbations in ASD astrocytes. GSEA revealed enhanced activity in processes related to proliferation and blood-brain barrier function, alongside suppression of mitochondrial respiration and metabolic pathways (Fig. 1E). KEGG analysis further indicated dysregulation in inflammatory pathways, glucose metabolism, oxidative phosphorylation, and neurodegeneration-related signals (Fig. 1E). These profiles align with established signatures of stress-induced astrocytic remodeling in neurological disorders [42–44], suggesting that astrocytes in ASD undergo functional alterations consistent with a stress-related response.

Previous studies have demonstrated that stress-induced perturbations represent a critical mechanism that remodels the microenvironment and drives and amplifies neuronal pathological changes [45]. Moreover, cell-type proportion imbalances have recently been recognized as key pathological hallmarks of neurodevelopmental disorders [46, 47]. Therefore, although neuronal populations are identified as harboring the highest number of DEGs in ASD (Fig. 1C), the contribution of astrocytes to ASD pathophysiology should not be overlooked. In particular, elucidating the pathogenic mechanisms of ASD from the perspective of astrocyte stress responses is not only necessary but also represents an essential extension of the existing research framework.

### A novel stress-responsive astrocytes subpopulation in ASD pathophysiology

**Fig. 2:**
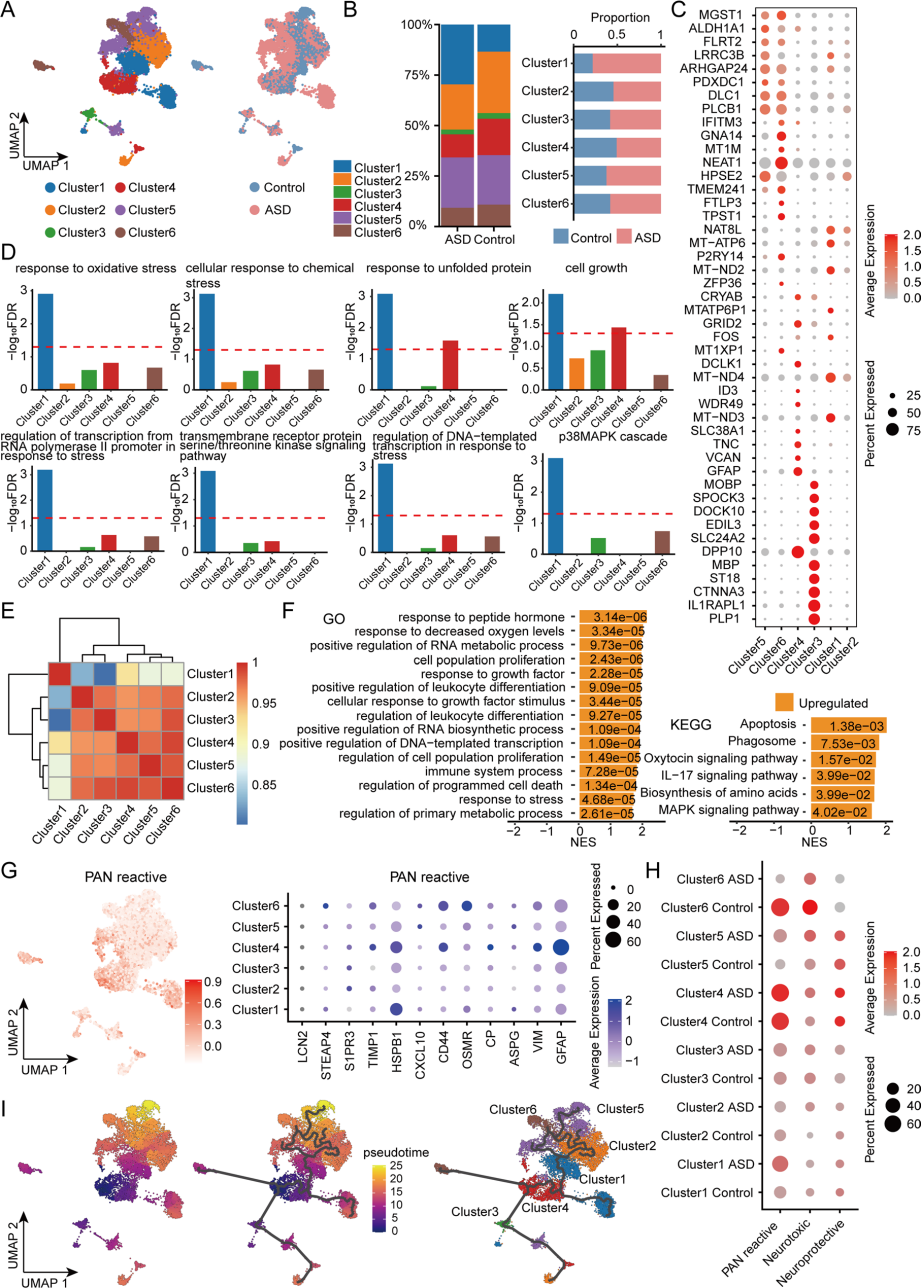
snRNA-seq identifies SRAs in human cortex.** (A)** Unsupervised clustering of astrocytes reveals six transcriptionally distinct subpopulations. **(B)** Cellular proportions of each astrocyte subpopulation across ASD and controls, visualized by grouped (left) and proportional (right) stacked bar carts. **(C)** Dot plot displaying the average expression and proportion of cluster-specific markers across the six astrocyte subpopulations. Differential expression analysis was assessed using the Wilcoxon ranked-sum test in Seurat. **(D)** GO analysis of DEGs between subpopulations highlighting specific term enrichments for Cluster1. The red dashed line indicates the significant threshold (FDR = 0.05). **(E)** Heatmap illustrating the correlations of expression profile among subpopulations, identifying SRAs as the most distinct. **(F)** GO and KEGG enrichment analyses of DEGs in SRAs from ASD versus control comparisons. **(G)** Expression levels of the pan-reactive signature score (left) and the corresponding dot plot for selected pan-reactive markers (right). **(H)** Combinatorial scores for pan-reactive, neurotoxic, and neuroprotective gene signatures across astrocyte subpopulations in ASD and controls. **(I)** Developmental trajectory of astrocyte subpopulations inferred by Monocle3

To delineate stress-associated astrocyte heterogeneity in ASD, we performed unsupervised UMAP clustering of astrocytes, identifying six transcriptomically distinct subpopulations (Cluster1-6) present across ASD and controls (Fig. 2A-C). Notably, only Cluster1 was significantly expanded in ASD, while other clusters showed reduced or unaltered proportions, suggesting its specific pathological relevance (Fig. 2B). Go analysis revealed that Cluster1 was uniquely enriched in stress-responsive processes, including oxidative stress, chemical stress, the unfolded protein response, and RNA transcription, distinguishing it from subpopulations linked to synaptic, developmental, or inflammatory functions (Fig. 2D, Figure S2A; Results S2).

Based on this signature, we defined Cluster1 as “stress-responsive astrocytes (SRAs)”. Transcriptomic analysis demonstrated that SRAs exhibited the highest transcriptional heterogeneity (Fig. 2E). Differential expression analysis also revealed significant transcriptional alterations in SRAs in ASD (Figure S2B), with upregulated pathways involved in responses to hypoxia, growth factors, and peptide hormones, alongside activation of apoptosis, autophagy, and IL-17 signaling (Fig. 2F, Figure S3). Critically, when evaluating SRAs reactivity states using gene set scoring and Gaussian mixture modeling, we discovered that although SRAs from ASD donors showed elevated pan-reactive marker expression, they displayed low scores for both neurotoxic A1 and neuroprotective A2 signatures, indicating a non-polarized reactive phenotype distinct from classical dichotomies (Fig. 2G-H, Figure S4 A-C; Results S3). Pseudotime trajectory analysis further supported a potential developmental transition from a quiescent state (represented by Cluster4) toward the SRA phenotype in ASD (Fig. 2I). Together, these findings identify SRAs as a previously undefined, non-polarized, stress-responsive astrocyte subpopulation that emerges during an early stage of ASD pathogenesis and may contribute to disease-specific pathophysiology, representing a novel component of the astrocytic response in ASD.

### JUND orchestrates transcriptional dysregulation in ASD SRAs

**Fig. 3:**
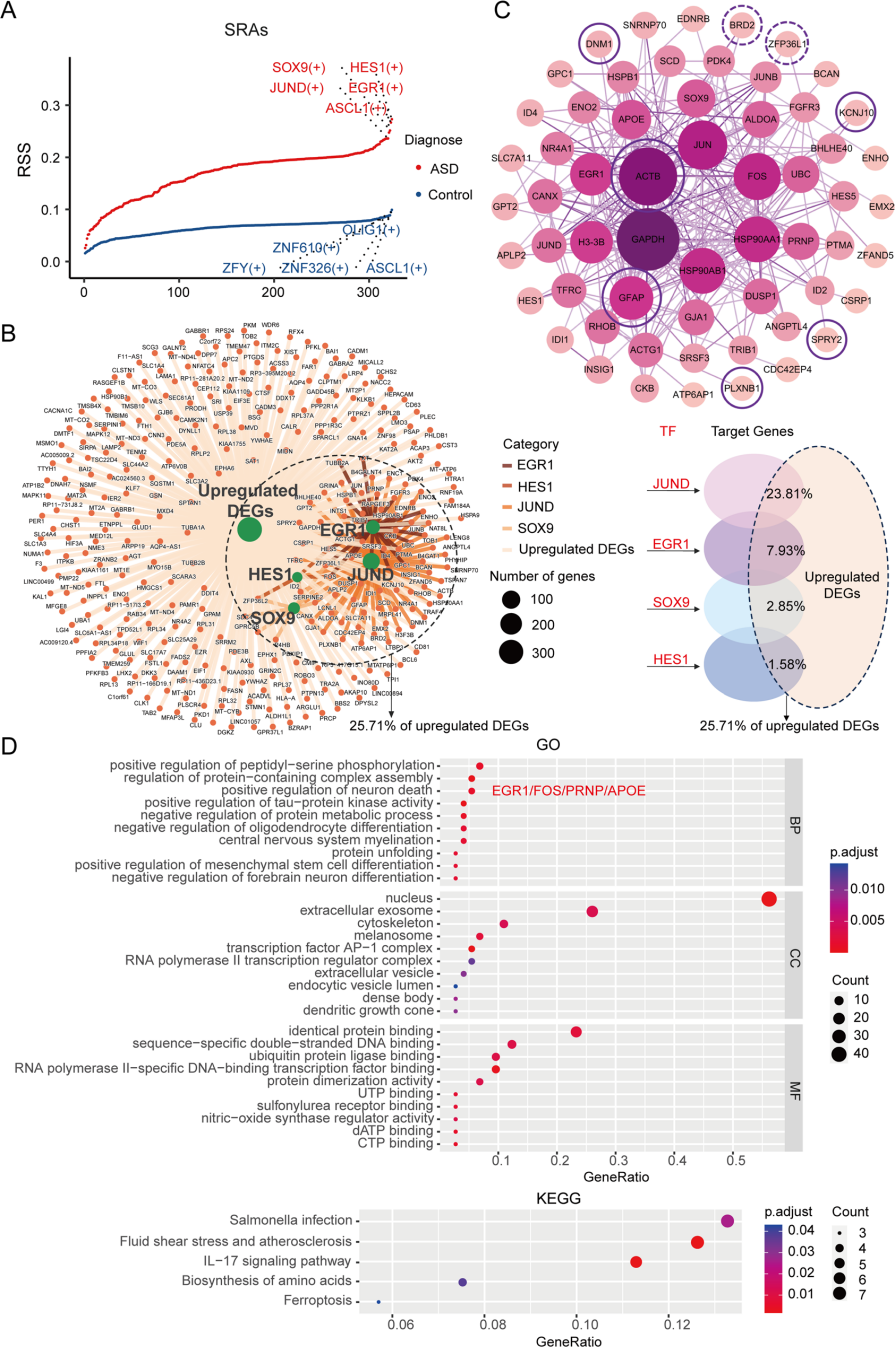
JUND is a key regulon coordinating a network of ASD risk genes.** (A)** Rank plot of top regulons in ASD and control, ordered by regulon specificity score (RSS) in astrocyte SRAs. **(B)** The co-regulation network illustrates the relationship between upregulated DEGs in ASD SRAs and the target gene sets of the top four regulons (JUND, EGR1, SOX9, HES1; left). Percent overlap between upregulated DEGs in SRAs and predicted regulons (right). These regulons together account for 25.71% of upregulated DEGs in astrocyte SRAs. **(C)** PPI network of JUND regulon predicted to regulate upregulated DEGs. Edge color intensity corresponds to the combined score, ranging from light (low confidence, 0) to dark (high confidence, 1). Node size and color (from small/light to large/dark) represent the degree of connectivity. Solid circles represent ASD risk genes from the SFARI database, and dashed circles indicate common ASD risk variants. **(D)** GO (top) and KEGG (bottom) enrichment analyses of JUND regulon predicted to regulate upregulated DEGs. Genes shown are associated with the enriched term “positive regulation of neuron death”

To uncover the molecular mechanisms driving ASD SRAs dysfunction, we employed pySCENIC to construct TF regulons across all six astrocyte clusters. This revealed divergent TF regulatory landscape, notably, SRAs exhibiting the most distinctive core TF regulons (Fig. 3A, Figure S5, Results S4). Specifically, SRAs from ASD donors showed heightened activation of gene regulatory network (GRN) linked to glial differentiation (SOX9 regulon) and stress response (JUND and EGR1 regulons), while control SRAs were enriched in developmental regulatory networks such as OLIG1 regulon (Fig. 3A). By integrating TF regulons with DEG data, we identified JUND as the dominant transcriptional regulator in ASD SRAs, governing 23.81% of upregulated genes, followed by EGR1 (7.93%), SOX9 (2.85%) and HES1 (1.58%; Fig. 3B). PPI analysis revealed that JUND target genes form a tightly connected network with JUND served as a central hub, interacting with direct TFs (e.g., JUN, FOS), indirect modulators (e.g., BHLHE40, ID proteins), and regulators of TF activity (e.g., HES1, DUSP1, TRIB1; Fig. 3C). Functional annotation of JUND target upregulated DEGs revealed significant enrichment in processes related to negative regulation of forebrain development, promotion of neuronal death, and pathways involved in IL-17 signaling and ferroptosis (Fig. 3D). Notably, the JUND regulon was significantly enriched with high-confidence ASD risk genes from the SFARI database, including *ACTB*, *GFAP*, *KCNJ10*, *DNM1*, *SPRY2*, and transcriptional regulators like *BRD2* and *ZFP36L1* (Fig. 3C), which have been implicated in synaptic function, neuronal excitability, and glia-neuron communication [48–51]. Taken together, our results position JUND as a master regulator of SRAs transcriptional reprogramming in ASD, potentially driving a stress-induced molecular cascade that contributes to cortical dysfunction and neuronal vulnerability via dysregulation of key ASD-related genetic networks.

### Enhanced gap junction communication mediates aberrant astrocyte-neuron communication in ASD

**Fig. 4:**
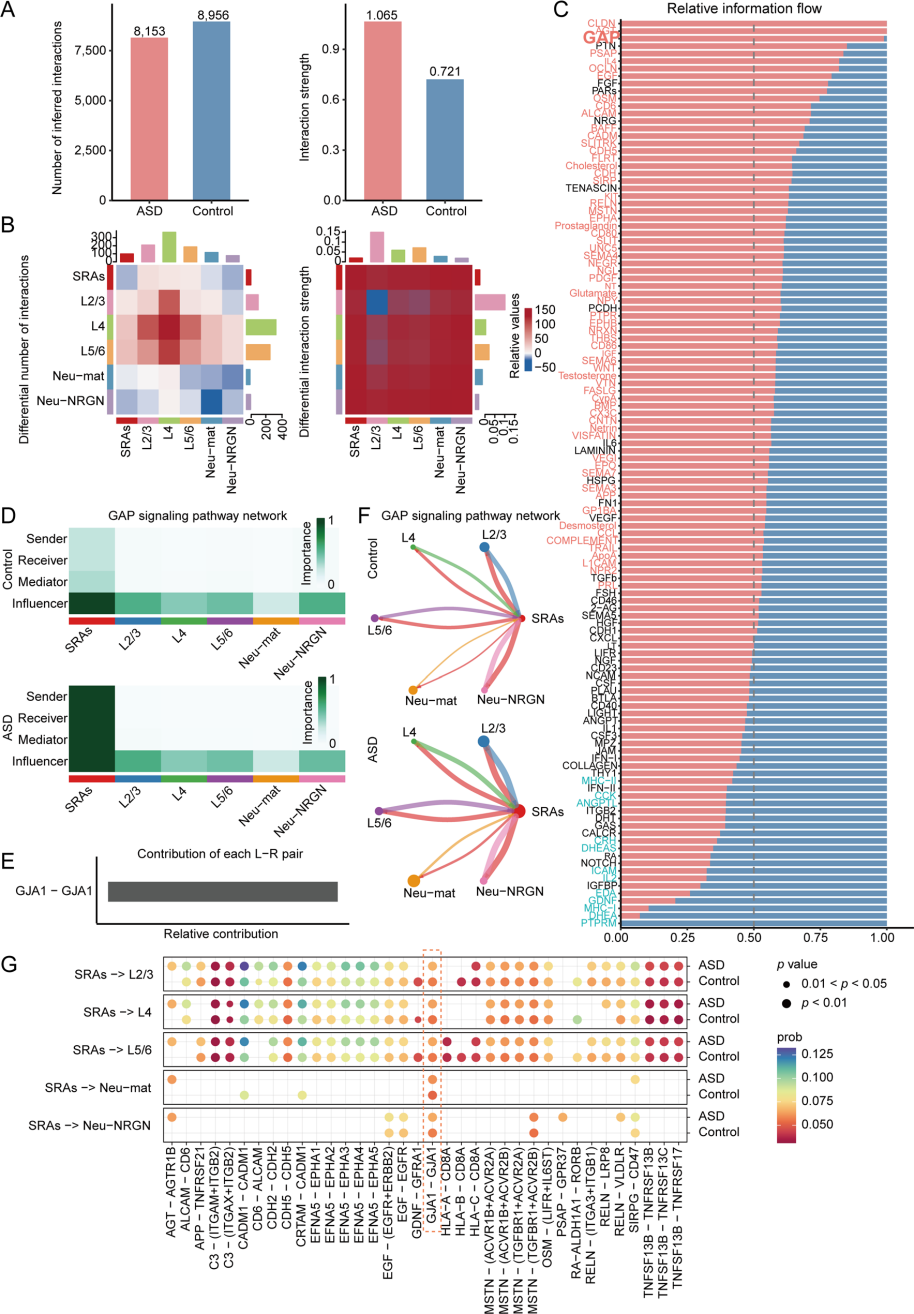
CellChat analysis of altered intercellular communication in the ASD cortex.** (A)** A comparison of the predicted number and strength of ligand-receptor interactions between SRAs and excitatory neuronal subpopulations in ASD versus control groups, analyzed by CellChat. **(B)** Heatmap depicting the CellChat-predicted differences in number and strength of ligand-receptor interactions between SRAs and five excitatory neuronal subpopulations in ASD compared to controls. **(C)** Relative information flow of signaling pathways between SRAs and excitatory neuronal subpopulations in the PFC of ASD and control group. Pathways with greater information flow in ASD are highlighted in red, those greater in controls are in blue, and those with no significant differences are in black. **(D)** Heatmaps showing the relative signaling roles of each cell type as the sender, receiver, mediator, and influencer in ASD and controls. **(E)** Relative contribution analysis of ligand-receptor pair identifies GJA1-GJA1 pair as the exclusive contributor to the GAP signaling pathway. **(F)** CellChat-generated netVisual circle showing the GAP signaling pathway communication probabilities between SRAs and five excitatory neuronal subpopulations in ASD and control groups. The size of the dots represents the number of cells, and the thickness of the lines represents the strength of intercellular communication. **(G)** CellChat-generated dot plot showing the significant ligand-receptor pairs between ASD and controls, contributing to the signaling from SRAs to other cell types. Dot color reflects communication probabilities, and dot size represents *p*-values computed using a one-sided permutation test

To elucidate the pathological crosstalk between SRAs and excitatory neurons in ASD, we performed a systematic cell-cell communication analysis at subpopulation resolution. We observed that, although the total number of inferred interactions was modestly reduced in ASD, the overall communication strength was significantly enhanced (Fig. 4A). Specifically, interactions between SRAs and neurons in cortical layers L2/3, L4, and L5/6 were both more numerous and stronger in ASD, whereas communications with Neu-mat and Neu-NRGN neuronal subpopulations, though fewer in number, were also intensified (Fig. 4B). Analysis of overall information flow further revealed a pronounced bias toward upregulation in ASD group (*n* = 62), with far fewer pathways exhibiting downregulated activity (*n* = 11; Fig. 4C). Among the most enhanced were CLDN, AGT, and GAP signaling pathways, while PTPRM, DHEA, and MHC-I were suppressed in ASD. These findings implied that these signaling pathways might constitute crucial cellular communication alterations between SRAs and excitatory neurons implicated in the pathogenesis of ASD. Furthermore, we found that SRAs served as the dominant sender of GAP signaling in both ASD and controls, with this regulatory role being substantially strengthened under ASD conditions (Fig. 4D). Within the GAP signaling, the GJA1-GJA1 homotypic interaction represented the sole and predominant ligand-receptor pair, exerting the strongest effect on signaling cascade strength (Fig. 4E). Consistently, outgoing communication from SRAs to multiple excitatory neuron subpopulations via GJA1-GJA1 was significantly elevated in ASD (Fig. 4F, G). Collectively, these results indicated that GAP signaling pathway mediates enhanced communication between ASD SRAs and neurons through GJA1-GJA1 interactions. Given that *GJA1* is a direct transcriptional target of JUND (Fig. 3C) [52], the master regulator of SRAs reprogramming, these results support a model in which JUND-driven upregulation of *GJA1* potentiates gap junction-mediated astrocyte-neuron communication, thereby contributing to network dysfunction in the ASD cortex.

### JUND-driven astrocytic dysfunction promotes neuronal apoptosis via gap junction signaling

**Fig. 5:**
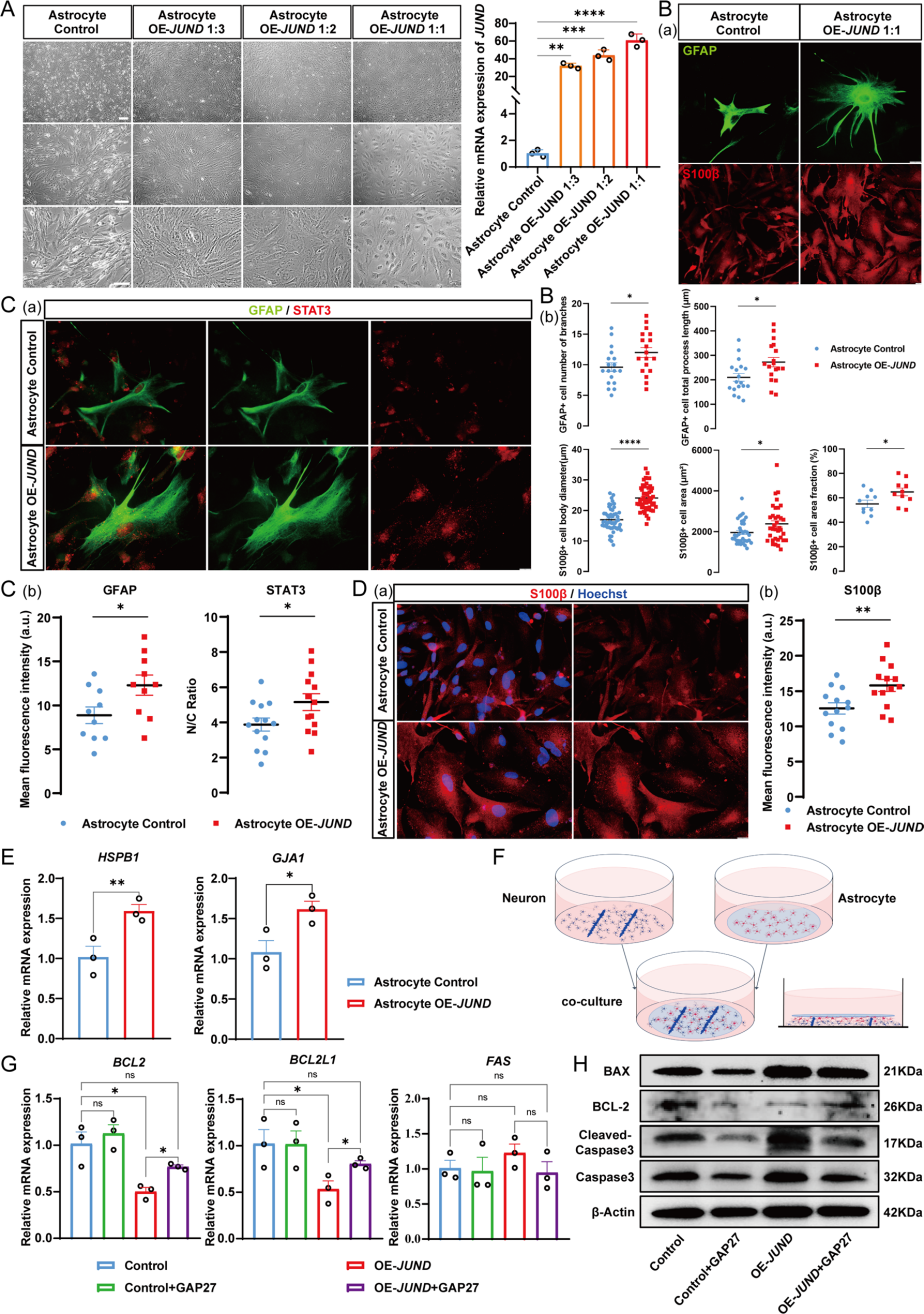
*JUND* overexpression in astrocytes primes co-cultured neurons for apoptosis. **(A)** Cellular morphology and molecular validation of astrocytes following overexpression of *JUND* (OE-*JUND*). Bright-field images of control (infection with an empty vector lentivirus) and OE-*JUND* (infected with a *JUND*-overexpressing lentivirus) astrocytes (left). Quantitative analysis of *JUND* mRNA levels (right). The ratio refers to that of the fresh virus volume to the culture medium volume. Scale bar, 0.2 mm. **(B)** Morphological analysis of GFAP-positive (green) and S100β-positive(red) astrocytes. **(a)** Representative images of astrocytic morphology. Scale bar, 20 μm. **(b)** Quantitative analysis of GFAP+ cell total process length (µm) and number of branches; and S100β + cell body diameter (µm), cell area (µm²), and area fraction. **(C)** Analysis of GFAP and STAT3 expression by immunofluorescence. **(a)** Representative images showing GFAP (green) and STAT3 (red) in control and OE-*JUND* astrocytes. Scale bar, 20 μm. **(b)** Quantitative analysis of GFAP mean fluorescence intensity and STAT3 nuclear-to-cytoplasmic (N/C) ratio in individual astrocytes. **(D)** Analysis of S100β expression by immunofluorescence. **(a)** Representative images showing S100β (red) in control and OE-*JUND* astrocytes. Scale bar, 20 μm. **(b)** Quantification of S100β mean fluorescence intensity. **(E)** Quantitative analysis confirms that *JUND* overexpression significantly upregulates *HSPB1* and *GJA1* expression in astrocytes (*n* = 3). **(F)** Schematic diagram of a direct contact co-culture model between neurons and astrocytes. **(G)** Relative expression of apoptosis-related genes ( *BCL2*, *BCL2L1*, *FAS*) in co-cultured neurons across the indicated treatment groups (*n* = 3). **(H)** Representative western blots showing the expression levels of key apoptosis-related proteins (BAX, BCL-2, Cleaved Caspase-3, Caspase-3) in co-cultured neurons, with β-Actin as the loading control. Data are presented as mean ± SEM; *p < 0.05, **p < 0.01, ***p < 0.001, ****p < 0.0001; ns, *p* > 0.05

To functionally validate the pathological effects of JUND-driven astrocytic dysfunction, we established a human iPSCs based co-culture model comprising purified astrocytes and neurons. Astrocytes were isolated via fluorescence-activated cell sorting (FACS) using established surface markers (Figure S6; Results S5) and subsequently infected with a *JUND*-overexpressing lentivirus (OE-*JUND*) or an empty vector lentivirus (Control). OE-*JUND* astrocytes exhibited pronounced morphological alterations characterized by increase in cell body diameter, process elongation, enhanced branching, and denser network formation compared to controls (Fig. 5A, B). This morphological shift was positively associated with the virus concentration, such that at a virus-to-medium ratio of 1:1, the transverse diameter of astrocytic cell bodies increased by 42% (*p* < 0.0001; Fig. 5A, B). Immunofluorescence analysis confirmed significant upregulation of reactive astrocyte markers (GFAP, S100β, VIMENTIN), enhanced nuclear translocation of STAT3 (Fig. 5C, D; Figure S7A) and elevated expression of the pan-reactive marker *HSPB1* in OE-*JUND* cells (Fig. 5E). Additionally, increased expression of inflammatory factors (*IL-6* and *IL-17A*) was observed (Figure S7B). These findings are consistent with the transcriptional patterns observed in ASD SRAs (Figure S7C). Q-PCR further revealed elevated expression of *GJA1* and pro-apoptotic associated genes *EGR1*, *FOS*, *PRNP*, and *APOE* in OE-*JUND* astrocytes (Fig. 5E; Figure S7D).

To determine whether JUND-mediated astrocytic changes affect neuronal survival through enhanced gap junction communication, we established direct-contact co-cultures between astrocytes and neurons (Fig. 5F) and specifically inhibited gap junctions using GAP27. After 24 h of co-culture with OE-*JUND* astrocytes, neurons exhibited significantly reduced expression of anti-apoptotic genes *BCL-2* and *BCL2L1*, while pro-apoptotic *FAS* expression remained unchanged (Fig. 5G). Western blot analysis confirmed decreased BCL-2 protein, increased BAX and cleaved caspase-3 levels (Fig. 5H; Figure S7E). Annexin V-FITC staining also revealed substantially enhanced neuronal apoptosis (Figure S7F). Importantly, these pro-apoptotic effects were abolished by GAP27 treatment, which alone had no significant impact on control co-cultures (Fig. 5G, H; Figure S7E, F). These results demonstrate that *JUND* overexpression drives astrocytes toward a pro-apoptotic state and that JUND-mediated neuronal death requires functional gap junction communication, revealing a mechanism by which stress-responsive astrocytes may contribute to neuronal vulnerability in ASD.

### Astrocyte-specific JUND overexpression recapitulates ASD-like behaviors in mice

**Fig. 6:**
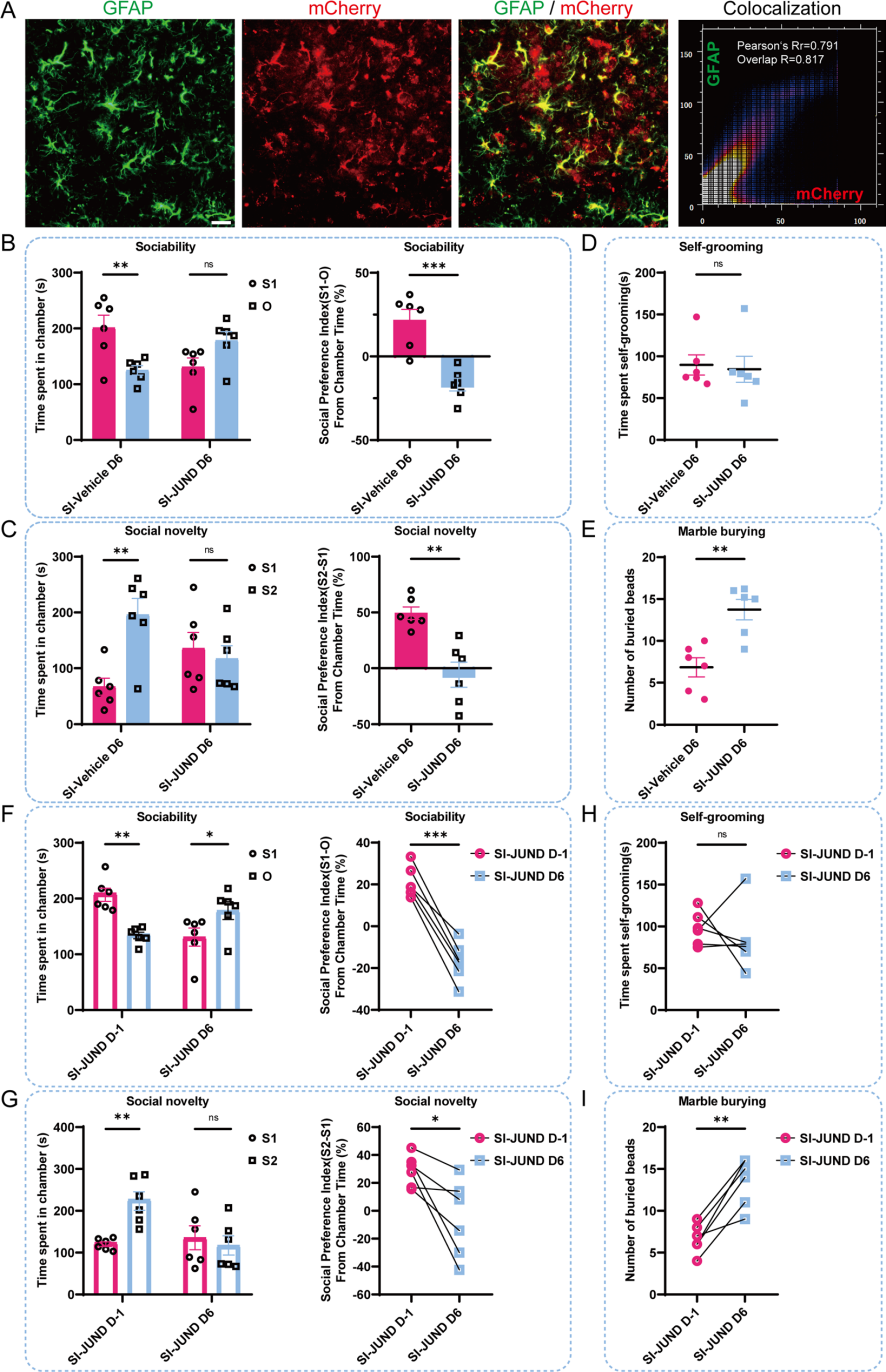
Astrocytic *JUND* overexpression induces ASD-like behavioral deficits in mice.** (A)** Colocalization analysis of GFAP and mCherry. Representative images of transfected mCherry (red)-labeled cells co-localized with GFAP (green) expressed astrocytes in mPFC. Scale bar: 20 μm. The colocalization panel (far right) depicts pixel overlap between GFAP and mCherry signals. Quantification of colocalization is shown in the scatter plot, with Pearson’s correlation coefficient (Rr = 0.791) and Overlap coefficient (*R* = 0.817) indicating a high degree of colocalization between GFAP and mCherry. **(B-E)** Behavioral assessments of mice at day 6 (D6) after treatment with SI-Vehicle or SI-JUND. **(B)** Sociability test. Total interaction time with novel mouse (S1) or object (O) during the three-chamber test (left). Social preference index in the stage of sociability (right). **(C)** Social novelty test. Time spent in the chamber with a familiar mouse (S1) versus a novel mouse (S2) (left). Social preference index in the stage of social novelty (right). **(D)** Representative results in the self-grooming test. **(E)** Representative results in the marble burying test. **(F-I)** Longitudinal behavioral analysis of mice before (Day-1, D-1) and after (Day6, D6) SI-JUND treatment (within-subject comparison) (*n* = 6). Data are presented as mean ± SEM; *p < 0.05, **p < 0.01, ***p < 0.001; ns, *p* > 0.05. SI-Vehicle, stereotaxic injection vehicle (PLV3-CMV-mCherry) and glycine; SI-JUND, stereotaxic injection JUND (PLV3-CMV-mCherry-*JUND*) and glycine

To investigate whether astrocytic JUND activation contributes to ASD-like behaviors, we stereotactically delivered *JUND*-overexpressing plasmid (SI-JUND) into mPFC astrocytes of C57BL/6N mice and assessed behavioral outcomes (Figure S8A). To confirm the astrocyte-specific targeting, we observed strong co-localization of mCherry with GFAP-positive astrocytes but not with NeuN-positive neurons (Fig. 6A; Figure S8B). Next, social ability and repetitive behaviors of the mice were evaluated before and on the 6th day post-injection (Figure S8A). In the three-chamber social test, SI-Vehicle mice showed a significant preference for interacting with the novel mouse (S1) over the object. In contrast, SI-JUND mice exhibited no significant preference between the object and the novel mouse, and their social index was significantly reduced, indicating impaired social ability (Fig. 6B). Furthermore, when presented with a second novel mouse (S2), SI-JUND mice failed to demonstrate the expected social novelty preference (Fig. 6C). These social deficits were accompanied by increased repetitive behaviors, as evidenced by enhanced marble-burying activity though self-grooming remained unchanged (Fig. 6D, E). Comparative analysis of pre- and post-injection behavioral profiles confirmed significant deterioration in social interaction, social novelty recognition, and repetitive behaviors following *JUND* overexpression (Fig. 6F-I; Figure S8C). Collectively, these findings demonstrate that specific JUND activation in mPFC astrocytes is sufficient to elicit core ASD-like phenotypes in mice, providing compelling evidence for its pathogenic role in ASD-related behavioral abnormalities and identifying astrocytic JUND activation as a potential therapeutic target.

## Discussion

Research into ASD has traditionally been constrained by a predominantly neurocentric framework, limiting comprehensive exploration of specialized glial subpopulations and their potential contributions to disease mechanisms [53, 54]. While accumulating evidence indicates significant functional heterogeneity among astrocytes in various neurodevelopmental contexts [55, 56], the existence of distinct, disease-specific astrocyte states actively contributing to ASD pathogenesis has remained an unresolved question. Through integrated snRNA-seq analysis of cortical tissues from a well-characterized ASD cohort, we have identified a previously unrecognized astrocyte subpopulation, SRAs, which exhibit a unique transcriptional signature enriched for stress response pathways and show disease-specific enrichment patterns. Furthermore, we delineate a JUND-mediated molecular cascade through which SRAs drive neuronal dysfunction, thereby establishing a novel astrocyte-dependent pathogenic pathway in ASD. Our findings not only reveal a novel cellular mechanism underlying ASD pathophysiology but also provide a framework for understanding how astrocyte heterogeneity may contribute to disease-specific phenotypes in neurodevelopmental disorders.

The identification of SRAs represents a significant conceptual advance beyond the traditional binary classification of reactive astrocytes into A1 or A2 states that has dominated the glial pathology field [57, 58]. While previous studies have documented generalized astrocyte reactivity in ASD postmortem brains [59], our detailed snRNA-seq analysis reveals a more complex and heterogeneous landscape of astrocyte responses in the disorder. SRAs emerge as a transcriptionally distinct entity, specifically enriched for stress-responsive pathways rather than classical neuroinflammatory signatures typically associated with A1/A2 polarization. The strategic positioning of SRAs at the earliest pseudotime transition positions them as a cellular interface linking environmental stressors to ASD pathology, providing a mechanistic basis for the established connection between early-life stress and disease exacerbation while reinforcing emerging concepts of astrocyte heterogeneity [60–62]. The specific expansion of SRAs in ASD tissues indicates they may represent a maladaptive response unique to the ASD molecular environment, possibly reflecting an interaction between genetic vulnerability and environmental stressors.

Our identification of JUND as the master regulator of the SRAs phenotype offers crucial insights into the molecular logic of astrocyte dysfunction in ASD. JUND, a component of the AP-1 transcription factor complex, has been previously implicated in various cellular stress responses [63, 64], but its specific role in astrocyte pathophysiology in neurodevelopmental disorders remained largely unexplored. The significant overlap between JUND-target genes in SRAs and established ASD risk genes creates a compelling molecular framework which environmental stressors, through JUND activation, may disrupt neurodevelopmental programs governed by genetic susceptibility factors [65], suggesting a point of convergence where diverse risk factors funnel into a common pathological pathway. Supporting this, we find that *JUND* mRNA is elevated in SRAs from ASD donors, and that *JUND* over-expression alone recapitulate the molecular features of SRAs. Complementing these human data, our in vivo experiments demonstrate that JUND activation in mPFC astrocytes is sufficient to recapitulate core ASD-like behavioral phenotypes. Together, these findings position JUND as a strategically positioned therapeutic entry point for disrupting the stress-to-pathology continuum in ASD.

The translational impact of this JUND-driven program materializes through its specific disruption of neuron-glia communication, which we have delineated as a coherent JUND-GJA1-GAP signaling axis. In this pathway, JUND directly upregulates *GJA1*, consequently enhancing gap junction-mediated communication between astrocytes and neurons. This finding is particularly significant in light of previous research showing that astrocytic gap junctions are crucial for maintaining synaptic homeostasis and that their dysregulation can contribute to network abnormalities in neurodevelopmental disorders [66, 67]. The reversal of neuronal apoptosis by GAP27 provides direct causal evidence that this communication pathway becomes maladaptive in the disease context. This dual nature of gap junction signaling, which serves essential physiological functions yet becomes pathologically detrimental upon dysregulation, aligns with emerging evidence across neurological conditions and underscores the critical importance of context-dependent interpretation of intercellular communication mechanisms in disease pathogenesis [68, 69]. Beyond the GJA1 connection, our findings of JUND-mediated transcriptional regulation of *EGR1* reveals an additional layer of complexity in how astrocytic stress responses may impact neuronal function in ASD. *EGR1*, an immediate-early gene responsive to various stimuli including stress, has been previously demonstrated to regulate the expression of *SHANK3* [70], a critical synaptic scaffolding protein strongly implicated in ASD pathogenesis [71]. While our study specifically demonstrates the JUND-EGR1 regulatory relationship in astrocytes, the established link between EGR1 and SHANK3 expression provides a conceptual framework for understanding how stress-responsive pathways in glial cells might ultimately affect synaptic organization. This mechanism may represent one explanation for how diverse etiological factors, including environmental stressors acting through glial cells, can converge on common synaptic phenotypes observed in ASD. The convergence of stress-responsive pathways with genes regulating synaptic function in astrocytes represents a previously underappreciated layer of complexity in ASD pathophysiology that merits further investigation.

The pro-apoptotic mechanism of the JUND-GJA1-GAP axis likely involves sophisticated interplay between disrupted glutamate homeostasis and neuroinflammatory signaling, creating a synergistic toxic environment for vulnerable neurons. Our observation of concurrent *SLC1A2* downregulation and *IL-6/IL-17A* upregulation in *JUND*-overexpressing astrocytes reveals a coordinated excitotoxic and inflammatory insult that may selectively target specific neuronal populations (Figure S7B). This pattern resonates with previous reports of altered glutamate transporter expression in ASD brains [72] and elevated inflammatory markers in ASD patients [73, 74], but the novel aspect of our findings lies in positioning these established abnormalities within a specific astrocyte subpopulation and tracing their origin to a defined transcriptional regulator. The JUND-GJA1-GAP axis thus appears to function as a molecular amplifier, where stress signals trigger transcriptional changes that simultaneously create a toxic communication channel via enhanced gap junction coupling while disabling protective mechanisms through glutamate transporter downregulation. This dual disruption creates a perfect storm where both the release of toxic factors and the clearance of potentially harmful substances like extracellular glutamate are compromised, ultimately creating a hostile microenvironment for neuronal survival and proper function.

### Limitations

Several important considerations and future directions emerge from our study. First, whether the JUND-GJA1-GAP axis represents a convergent mechanism across diverse ASD etiologies remains an open question, as direct evidence from established ASD models is currently limited. Systematic validation in these models will be essential to determine if it operates as a common pathogenic node or a pathway selectively engaged in stress-associated endophenotypes. Second, the SRAs and their associated mechanisms were identified in a specific cohort and may represent one of several astrocyte-driven pathological pathways within the heterogeneous ASD landscape. This heterogeneity has implications for therapeutic development, suggesting that astrocyte-targeted interventions may need to be tailored to specific molecular subpopulations. Third, the JUND-GJA1-GAP axis represents a druggable target with particular relevance for individuals with stress-related symptom exacerbation or evidence of JUND pathway engagement, yet its application may be restricted to precision therapies guided by accurate molecular diagnostics. Finally, several mechanistic questions remain to be addressed. The upstream activators of JUND in the ASD context require further elucidation, and the relationship between SRAs and other glial cell types, particularly microglia, warrants future investigation given their known cross-talk in other neuropathological conditions.

## Conclusions

To sum up, by identifying the SRAs and delineating the JUND-GJA1-GAP axis, our work provides both a novel perspective for understanding ASD pathophysiology and specific, therapeutically accessible targets for intervention. Our findings underscore the importance of moving beyond neuron-centric models of ASD to incorporate the crucial contributions of glial cells, particularly specialized astrocyte subpopulations that may drive specific aspects of disease pathogenesis. Future research should focus on validating these mechanisms in larger cohorts, developing targeted modulators of this pathway, exploring their potential utility across the spectrum of neurodevelopmental disorders, and identifying additional astrocyte subpopulations that may contribute to the complex pathophysiology of ASD.

## Supplementary Information

Below is the link to the electronic supplementary material.

Supplementary Material 1

## Data Availability

All data associated with this study are present in the paper or the supplementary files.
