# Supplementary material for: JUND-driven stress-responsive astrocytes promote neuronal apoptosis via enhanced gap junction signaling in autism spectrum disorder
Source: Mol Autism. 2026 May 19;17:25. doi: 10.1186/s13229-026-00717-0 (PMC13245007; doi:10.1186/s13229-026-00717-0)
Supplement: Supplementary file 1 — Supplementary Material 1 [file 13229_2026_717_MOESM1_ESM.docx]

**SUPPLEMENTARY INFORMATION**

**JUND-driven stress-responsive astrocytes promote neuronal apoptosis via enhanced gap junction signaling in autism spectrum disorder**

Wang *et al.*

**Supplementary Results**

**1. Altered cellular composition and transcriptional profiles in ASD cortex**

Comparative analysis revealed significant differences in cellular composition between ASD and control groups. Specifically, we observed a marked enrichment of astrocytes, L2/3 excitatory neurons, and OPCs in ASD samples, while other cell types generally showed reduced proportions or non-significant changes (Figure 1A, B). Statistical quantification confirmed that only the increase in astrocytes reached statistical significance (*p*<0.05), whereas other cellular alterations did not (Figure 1B).

Analysis of DEGs further revealed cell-type-specific transcriptional dysregulation in ASD. Among neuronal subpopulations, all classes except Neu-NRGN exhibited a transcriptional enhancement phenotype (Figure 1C). Neu-NRGN contained the highest number of DEGs, predominatly downregulated, followed by Neu-mat. In contrast, non-neuronal cells, including astrocytes, OPCs, oligodendrocytes, and endothelial cells, displayed consistent transcriptional downregulation (Figure 1C). Of particular interest, astrocytes exhibited a substantial number of DEGs in ASD compared with controls (Figure 1C). Alongside these broad transcriptional alterations in astrocyte-specific genes, we detected significant upregulation of the reactive astrocyte markers *HSPB1* and *SOX9* in ASD samples (Figure S1A-C). These findings suggest widespread dysregulation of cortical gene expression in ASD, particularly in astrocytes, with potential implications for stress-related mechanisms in the pathogenesis of the disorder.

**2. Distinct astrocyte subpopulations exhibit altered molecular pathways in ASD**

We identified six astrocyte subpopulations with distinct molecular signatures. Among these, Cluster6 was notable for its strong association with metal ion homeostasis (e.g., zinc, copper, and iron; Figure S2A) and for harboring the greatest number of DEGs in ASD (Figure S2B). Functional enrichment analysis of these DEGs revealed a concerted downregulated of inflammatory and defense responses. Conversely, functions governing synapse assembly and filopodium formation were significantly upregulated (Figure S3). At the signaling pathway level, we observed a suppression of the TGF-beta signaling and Cytokine-cytokine receptor interaction pathways, alongside an activation of the ErbB and cAMP signaling pathways (Figure S3). Cluster4, which was identified as a putative initial state in our pseudotime analysis (Figure 2I), is inferred to represent astrocyte precursor cells characterized by robust migratory capabilities (Figure S2A). In the context of ASD, this cluster displayed a predominant downregulation of broad metabolic processes (Figure S3). The transcriptional profile of Cluster2 highlighted its specific role in synapse formation, synaptic vesicle exocytosis, and axon development (Figure S2A). In ASD, this subpopulation exhibited severe mitochondrial impairment, evidenced by the significant downregulation of critical functions including mitochondrial ATP synthesis coupled electron transport, oxidative phosphorylation, and the respiratory electron transport chain (Figure S3). Cluster3 was annotated as potentially representing fibrous astrocytes, based on its functional enrichment in axon assembly, myelin formation, and oligodendrocyte differentiation (Figure S2A). In ASD, this cluster showed suppression of fundamental cellular processes such as protein folding and nuclear chromosome segregation. In contrast, the Oxytocin signaling pathway and the Gap junction pathway were notably upregulated (Figure S3). Finally, Cluster5 demonstrated the least transcriptional heterogeneity and the fewest DEGs among all subpopulations (Figure 2E, Figure S2B). Its defining functions centered on the regulation of immune cells, including macrophages, T cells, and monocytes (Figure S2A), suggesting a specialized role in neuro-immune crosstalk. In ASD, this cluster activated programs related to the regulation of protein stability, immune and defense responses, and cellular stress responses (Figure S3).

**3. Heterogeneous polarization of astrocyte subpopulations in ASD**

Astrocytes can polarize into two primary states: a neurotoxic/pro-inflammatory (A1) phenotype and a neuroprotective/anti-inflammatory (A2) phenotype. While this dichotomous framework does not fully capture the phenotypic diversity of astrocytes, it provides a basis for understanding their reactive states in central nervous system disorders. Studies have reported elevated astrocyte reactivity in the cerebral cortex of individuals with ASD; however, their polarization status and whether different functional subpopulations exhibit consistent polarization directions remain unclear. We performed a preliminary investigation by profiling reactivity marker genes across astrocyte subpopulations, which identified Cluster4 and Cluster6 as also highly reactive (Figure 2G, H). We further applied a Gaussian mixture model to stratify high-risk cells and evaluated their A1/A2-specific gene expression profiles (Figure S4A-C). The findings revealed an opposite polarization direction of Cluste4 between ASD and healthy controls: the ASD group tended toward a neurotoxic (A1) phenotype, whereas controls tended toward a neuroprotective (A2) phenotype (Figure S4C). In contrast, Cluster6 exhibited a distinct profile, with reduced pan-reactive and neurotoxic markers in ASD (Figure 2H), and displayed a polarization pattern opposite to that of Cluster4 (Figure S4C). Enriched for synaptogenesis genes in ASD, this subpopulation may represent an adaptive state of concurrent hyperactivation and reactive suppression, suggesting a shift from classical reactive pathways toward supporting synaptic remodeling and neuroprotection. Pseudotime trajectory analysis further positioned Cluster4 as an initial astrocyte state, potentially representing a precursor population poised for differentiation (Figure 2I). As the pathological trajectory progresses, Cluster1 (SRAs) emerges as a stress-responsive subpopulation significantly expanded in ASD, likely driving early neurotoxic processes. In contrast, Cluster6 exhibited a low reactivity signature and may function as a compensatory subpopulation at later stages (Figure 2I), potentially counterbalancing Cluster1-induced injury. Together, these three subpopulations delineate a dynamic pathological continuum-from an initial poised state (Cluster4) through a neurotoxic phase (Cluster1) to a potential compensatory response (Cluster6)-suggesting that astrocyte-mediated pathophysiology in ASD involves a temporal shift from early dysfunction to later structural and functional reorganization. Deciphering the regulatory logic of these astrocyte subsets will pave the way for targeted therapies.

**4. Analysis of TF activity in astrocyte subpopulations**

Analysis of TF activity revealed that SRAs exhibited the most pronounced disparity in the GRN between individuals with ASD and controls, with distinct core TFs for each group (Figure 3A). Cluster5 also showed marked differences, characterized by increased transcriptional activity of RUNX1 and NFIA in ASD individuals, alongside decreased activity of BRF2 and ESRRG (Figure S5). Notably, the top five active TFs in Cluster6 were entirely different between ASD and the control groups (Figure S5). Specifically, TEAD4, STAT5A, FOXO1, RFX2, and ARID3A were the most active TFs in ASD, whereas GLI2, BCL6, IRF1, ETV6, and PML were most active in controls. This divergent TF profile may may contribute to the substantial number of DEGs observed in this subpopulation (Figure S2B). In Cluster2, JDP2, EGR3, and HEY2 exhibited abnormal activation in ASD, while ZNF853, KLF16, and THRA displayed relatively decreased activity (Figure S5). In contrast, TF activity disparities in Cluster3 and Cluster4 were relatively modest. The top five TFs converged completely in Cluster3 and were largely consistent in Cluster4, diverging only in the heightened activity of ZNF281 in ASD and of ATF3 in controls (Figure S5).

**5. Differentiation of human astrocytes and neurons from iPSCs**

To investigate the effect of JUND activation in human glial cells on neuronal function, we firstly established a differentiation system capable of generating either neural cells or astrocytes from human iPSCs, as outlined in Figure S6A. Human iPSCs maintained characteristic undifferentiated morphology (Figure S6A). Neural differentiation was induced under defined conditions using a commercial neural induction system (STEMCELL TECHNOLOGIES). During this process, cells formed aggregates exhibiting typical EB morphology in suspension culture (Figure S6A). Following re-plating on Matrigel with neural induction medium for 12 days, EBs successfully differentiated into NPCs, as validated by expression of the NPC marker PAX6 (Figure S6B). NPCs were subsequently directed toward an astrocytic lineage through a combined induction and FACS strategy, yielding a highly pure population of astrocytes that robustly expressed the astrocytic markers S100β and GFAP (Figure S6B). For neuronal differentiation, NPCs were cultured in neural differentiation medium on a poly-L-ornithine/laminin-coated substrate. Within 10 days, the differentiated cells displayed characteristic neuronal morphology and were immunopositive for the neuronal marker Tuj1 (Figure S6B). With prolonged culture, neural processes extended progressively and functional network connectivity matured.


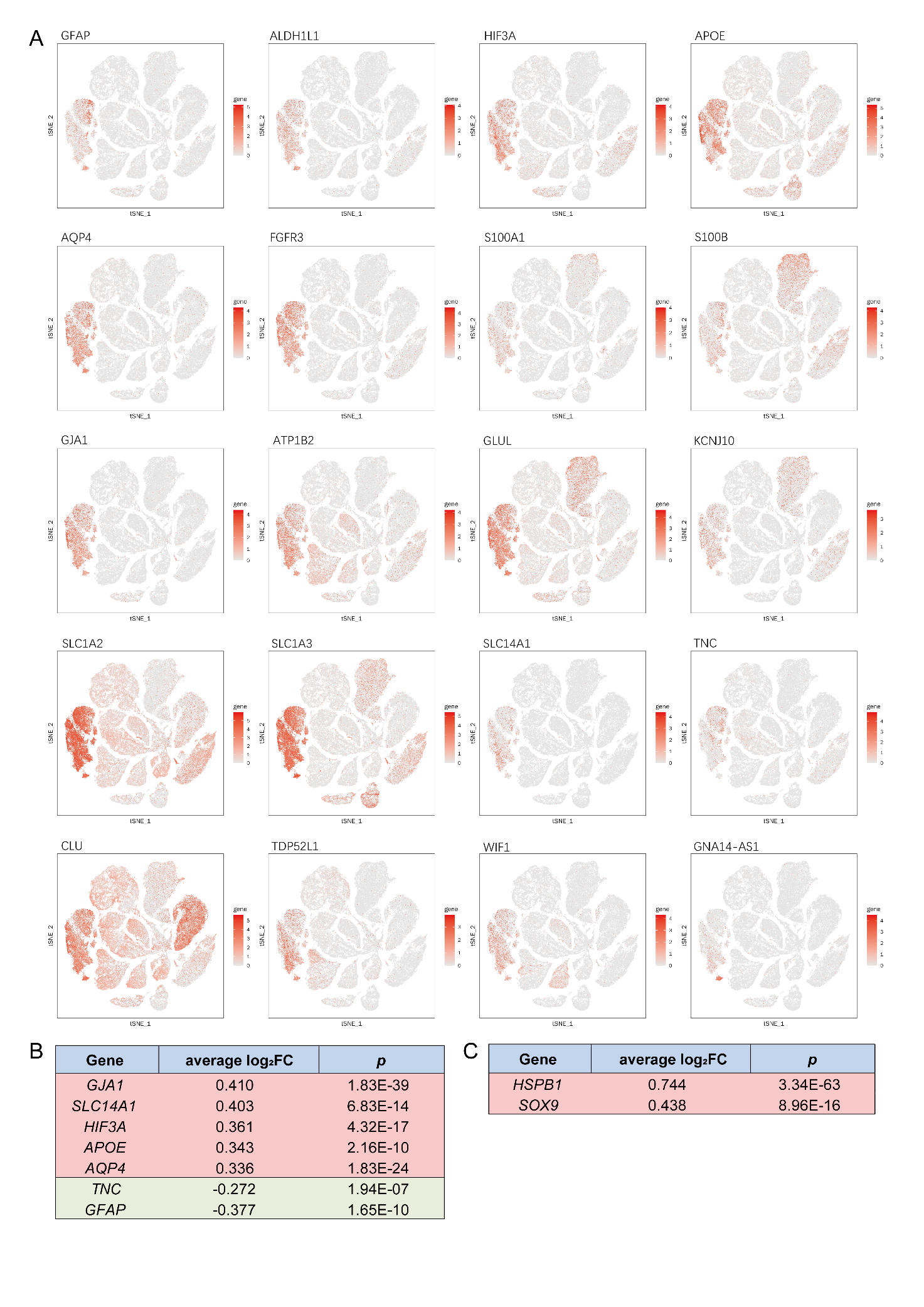


**Figure S1. The transcriptional landscape of astrocytes in ASD. (A)** Expression levels for canonical astrocytic markers in the snRNA-seq data. Differential expression level of astrocyte markers **(B)** and reactive astrocyte markers **(C)** in astrocytes between ASD and controls. FC, Fold-change. Upregulated genes, pink; downregulated genes, light green.


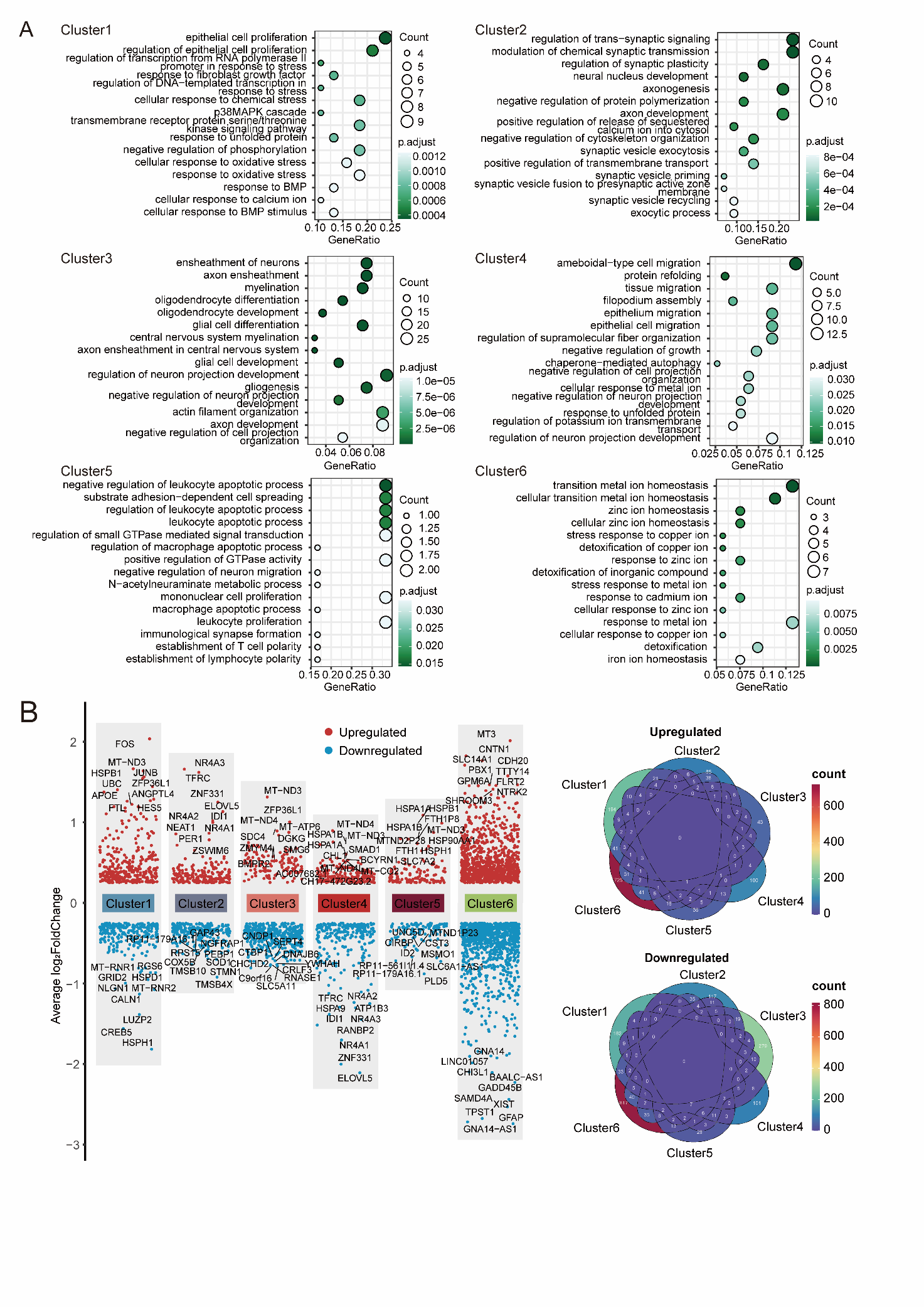
 **Figure S2. Functional and reactive heterogeneity among astrocyte subpopulations. (A)** Top 15 biological processes for each astrocyte subpopulation. **(B)** Multi-subpopulations volcano plots of DEGs of six astrocyte subpopulations (left); Venn diagram of the number of DEGs in different subpopulations (right).
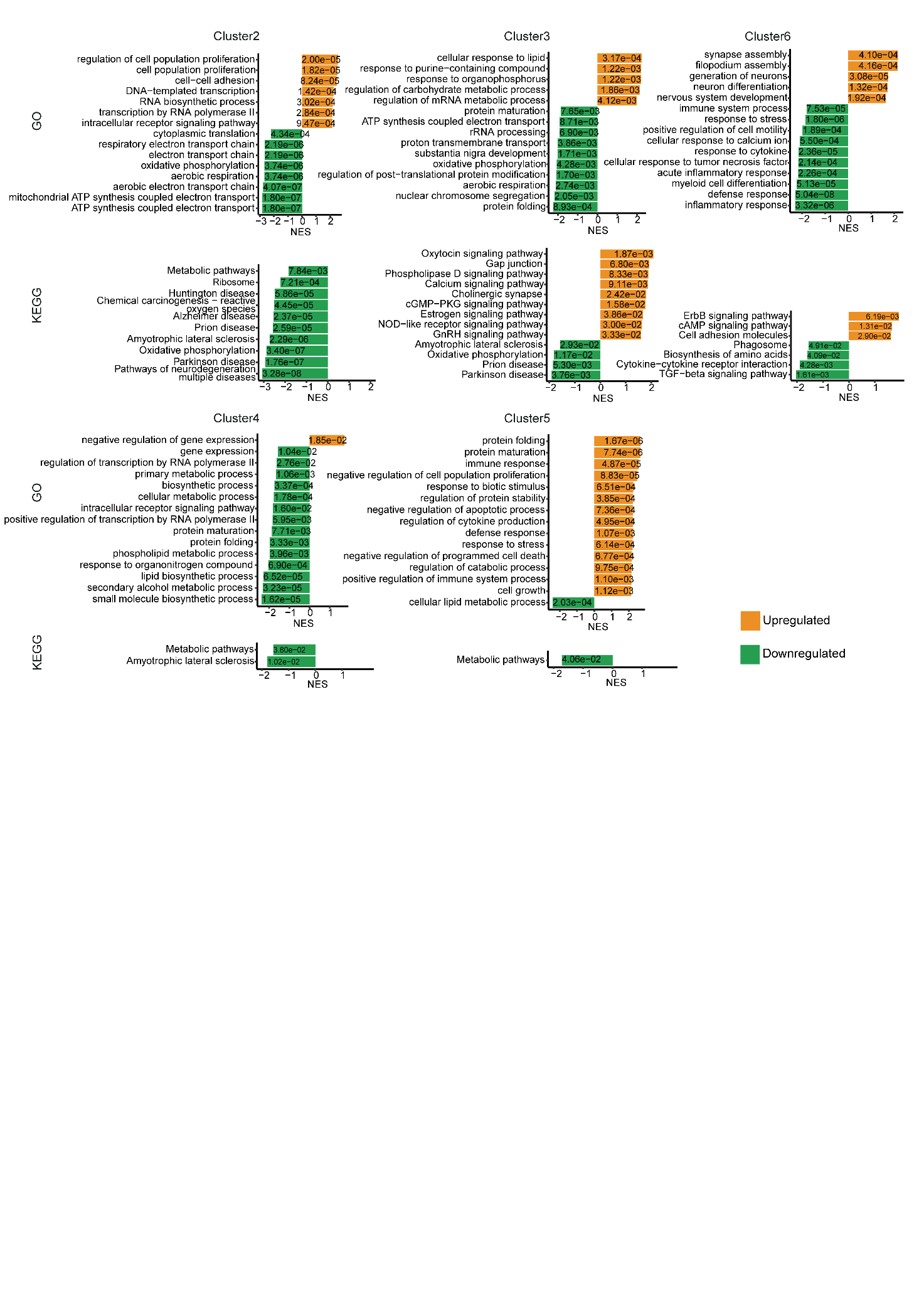


**Figure S3.** **Dysregulated function among astrocyte subpopulations in ASD.** GO and KEGG enrichment analyses of DEGs between ASD and controls in astrocyte subpopulations.


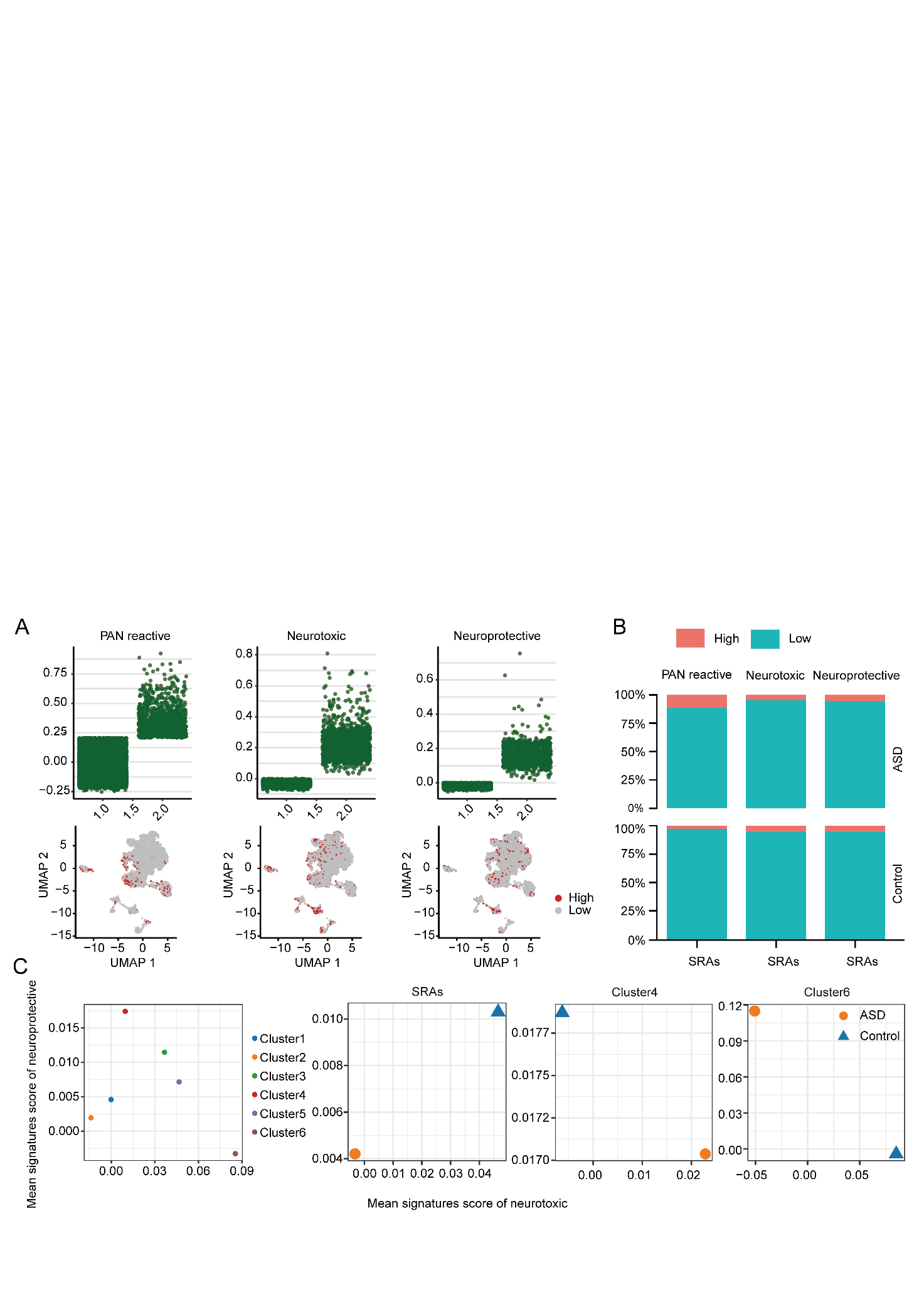


**Figure S4.** **Reactive changes of astrocytes. (A)** Stratification of patients into high-risk and low-risk groups using a Gaussian mixture model based on PAN reactive, neurotoxic, and neuroprotective scores (top); Distribution of samples based on PAN reactive, neurotoxic, and neuroprotective in the UMAP space, with red representing high reactivity ("High") and gray representing low reactivity ("Low") (bottom). **(B)** Bar chart shows the percentage of highly reactive cells of PAN reactive, neurotoxic and neuroprotective in SRAs. SRAs, stress-responsive astrocytes. **(C)** Scatter plot depicting the mean signature scores of neurotoxic (x-axis) versus neuroprotective (y-axis) across six subpopulations (left). Scatter plot showing the mean signature scores of neurotoxic (x-axis) versus neuroprotective (y-axis) in SRAs, Cluster4, and Cluster6 (right).


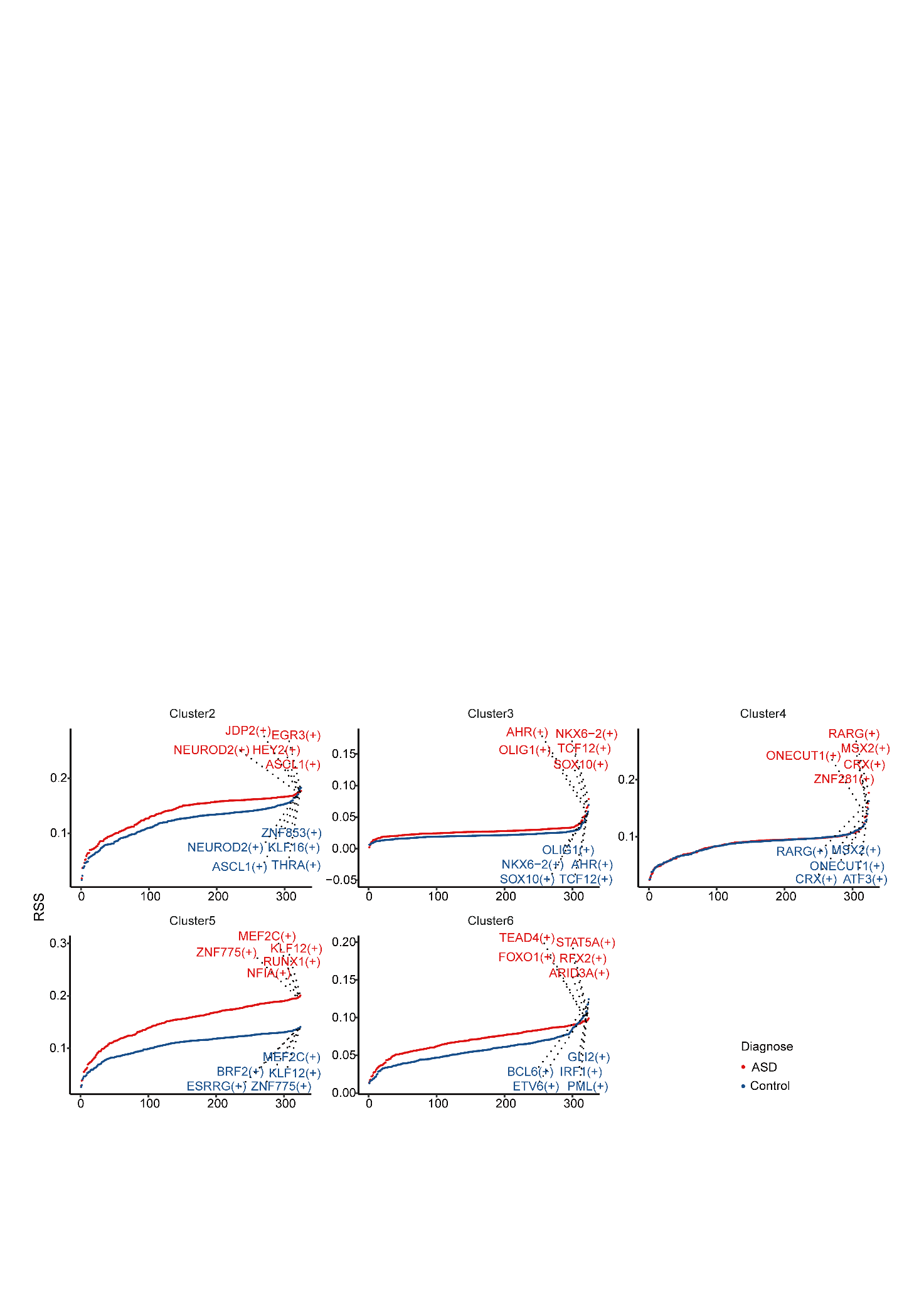


**Figure S5. Dysregulated transcriptional landscape in ASD astrocytes subpopulations.** Rank plot of ASD and control top regulons ordered by regulon specificity score (RSS) in cortical astrocyte subpopulations.


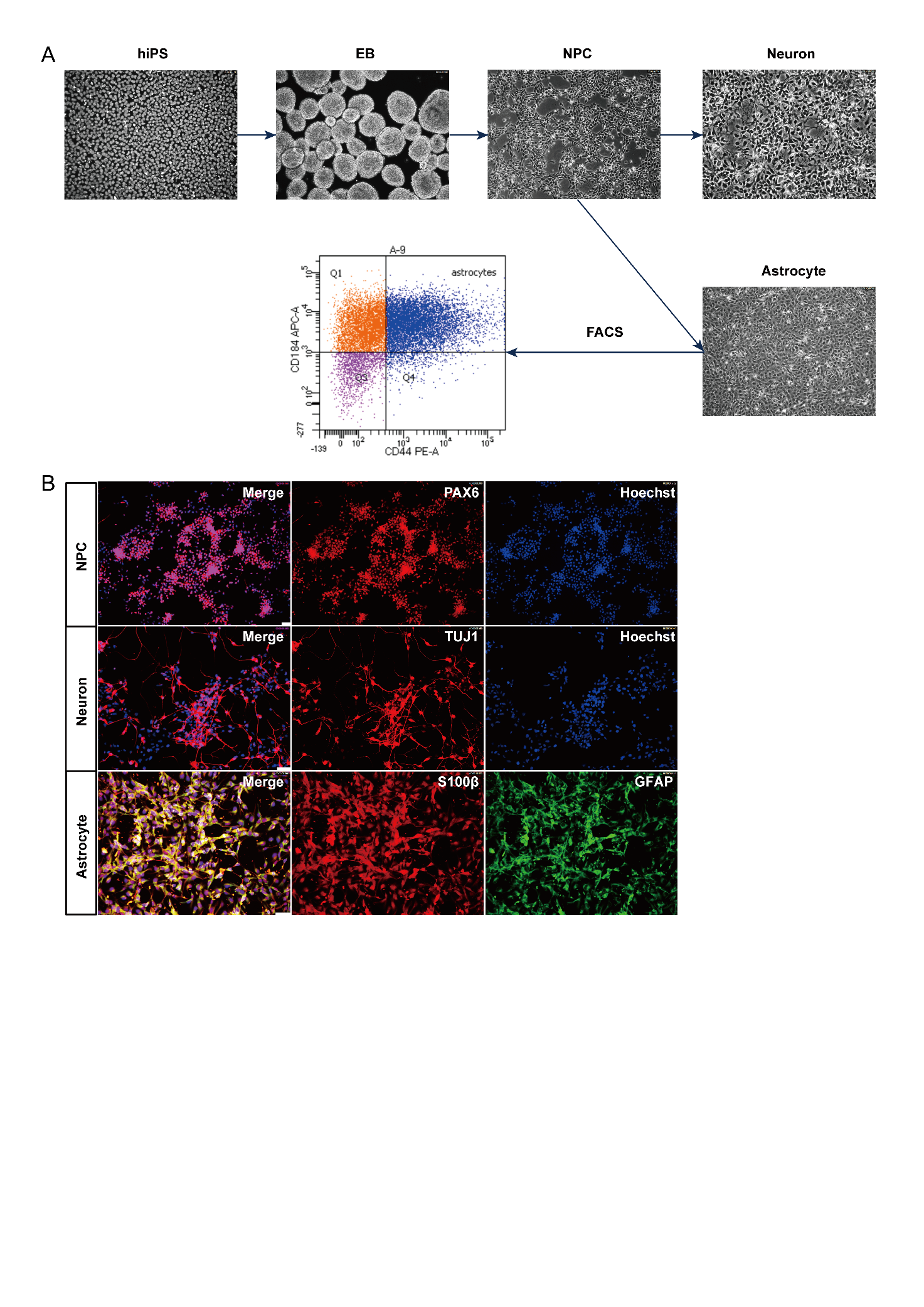


**Figure S6. Morphology and identification of human iPSCs differentiated cell. (A)** Schematic diagram illustrating the differentiation of human iPSCs into NPCs and their subsequent lineage specification. NPCs were directed toward either neuronal differentiation or subjected to fluorescence-activated cell sorting (FACS) using surface markers CD184 and CD44 to isolate an enriched astrocyte population. A representative flow cytometry scatter plot demonstrates the gated astrocyte population based on these specific markers. **(B)** Immunofluorescence analysis confirms the expression of stage-specific markers during differentiation. NPCs show positive staining for PAX6, while differentiated neurons express the neuronal marker TUJ1. Astrocytes exhibit co-localization of characteristic markers S100β and GFAP. Nuclei are stained with Hoechst. Scale bar, 50μm.


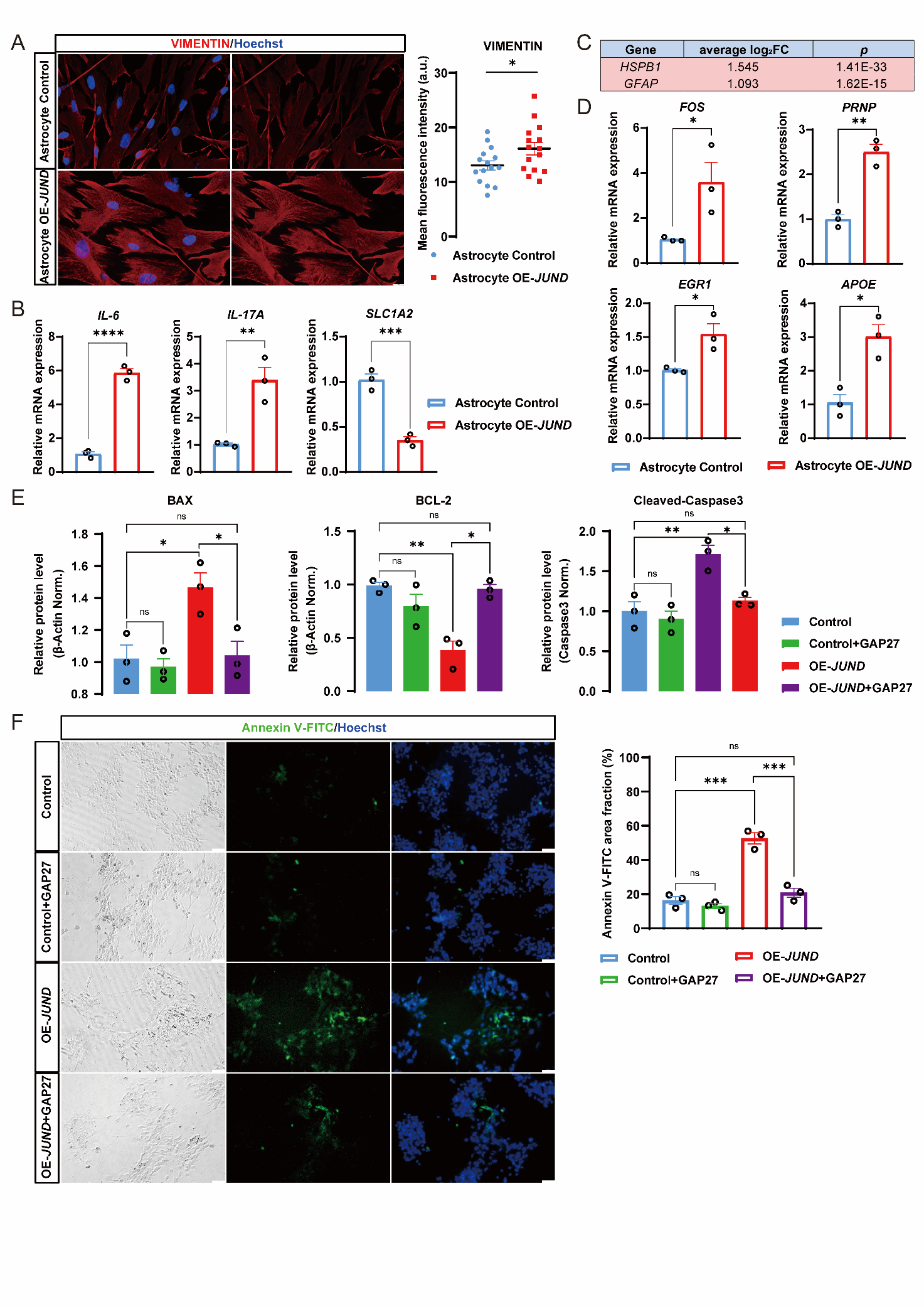
 **Figure S7. JUND modulates astrocyte function and primes neuronal apoptosis. (A)** Analysis of VIMENTIN expression by immunofluorescence. Representative images showing VIMENTIN (red) in control and OE-*JUND* astrocytes (left). Scale bar, 20μm. Quantification of VIMENTIN mean fluorescence intensity (right). **(B)** Relative expression of *IL-6*, *IL-17A*, and *SLC1A2* in control versus OE-*JUND* astrocytes (n=3). **(C)** Differential expression level of *HSPB1* and *GFAP* in SRAs between ASD and controls. FC, Foldchange. **(D)** Quantitative analysis demonstrates marked upregulation of *FOS*, *PRNP*, *EGR1,* and *APOE* following *JUND* overexpression in astrocytes (n=3). **(E)** Quantitative analysis of protein levels of BAX, BCL-2, and Cleaved-Caspase3 were measured by western blotting, normalized to β-Actin or Caspase3, and expressed as fold change relative to the Control group (n=3). **(F)** Annexin V-FITC staining for apoptosis in co-cultured neurons across treatment groups (left). Triple-panel images show bright-field (left), Annexin V-FITC (green, middle), and merged channels (right) of Annexin V-FITC (green) and Hoechst (blue). Scale bar, 50μm. The statistical graph presents the percentage of Annexin V-positive cells relative to total Hoechst-stained nuclei for each experimental group (right). Quantitative data are presented as mean ± SEM across groups (**p*<0.05, ***p*<0.01, ****p*<0.001).


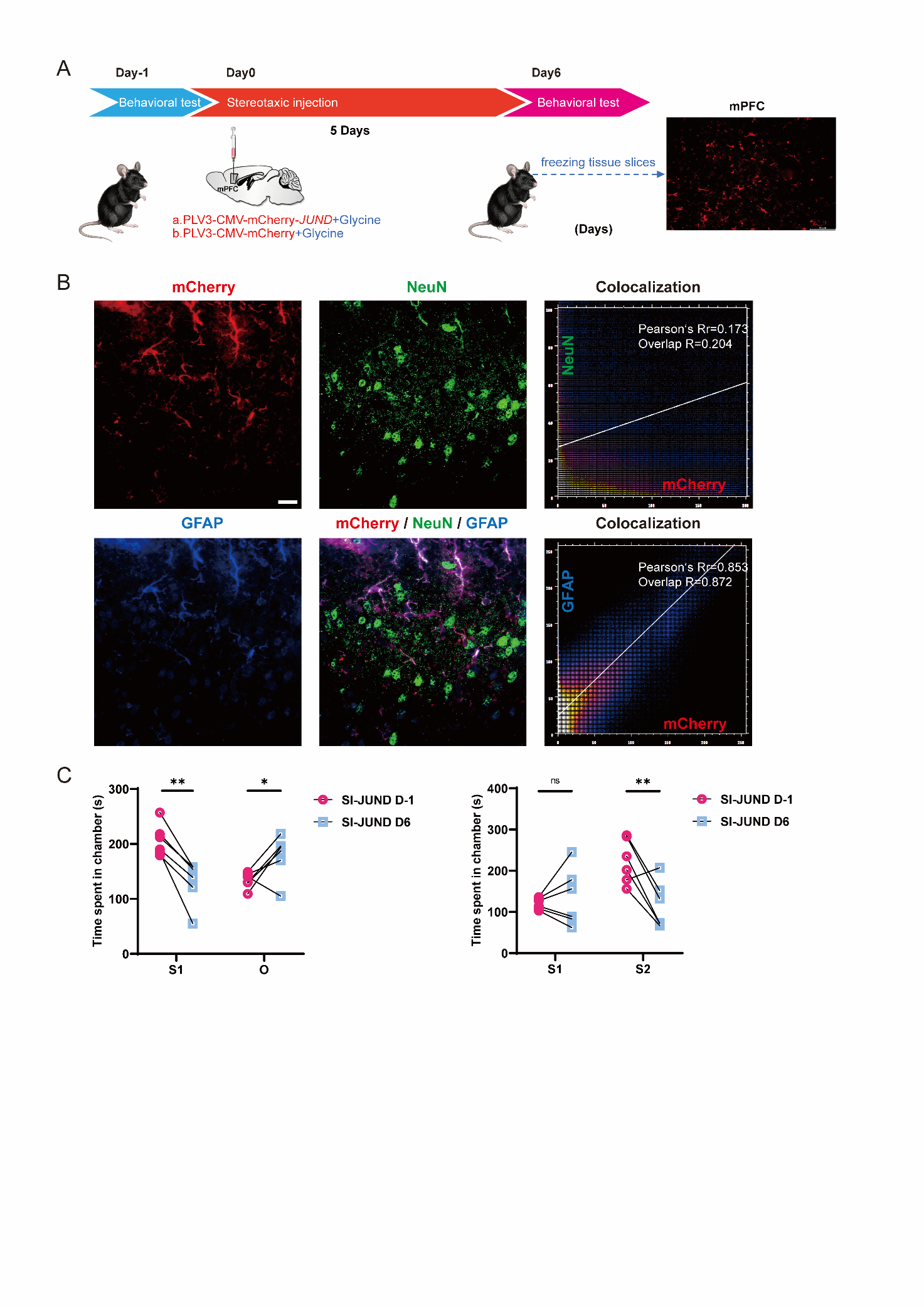
 **Figure S8. Schematic timeline and behavioral outcomes of medial prefrontal cortical JUND overexpression. (A)** Schematic diagram of the experimental timeline. Behavioral tests were first conducted at baseline (Day-1, D-1). On D0, gDAM mixture was stereotactically injected into the mPFC of mice. Two constructs were used: (a) gDAM mixture: PLV3-CMV-mCherry-*JUND* and glycine for *JUND* overexpression, and (b) gDAM mixture: PLV3-CMV-mCherry+glycine as the vehicle control. The same behavioral tests were repeated 6 days post-injection (D6), after which mPFC tissues were collected and processed for cryosectioning. A representative fluorescence image of the mPFC section is shown on the right. **(B)** Colocalization analysis of NeuN, GFAP, and mCherry. Left panels show representative fluorescence images of mCherry (red), NeuN (green), GFAP (blue), and the merged image (mCherry/NeuN/GFAP). Scale bar: 20 μm. Right panels depict colocalization analyses: scatter plots with quantification of pixel overlap between mCherry and NeuN (top) and between mCherry and GFAP (bottom). For mCherry/NeuN, Pearson's correlation coefficient (Rr=0.173) and Overlap coefficient (R=0.204) indicate negligible colocalization; for mCherry/GFAP, Pearson's Rr=0.853 and Overlap R=0.872 indicate strong colocalization. **(C)** Behavioral analysis of Sociability and social novelty preference. Time spent in the chamber containing a social conspecific (S1) versus an empty chamber (O), compared between pre-treatment (D-1) and post-treatment (D6) time points in the SI-JUND group (left). Time spent in the chamber with a familiar mouse (S1) versus a novel mouse (S2), compared between D-1 and D6 in the SI-JUND group (right). (n=6) Data are presented as mean ± SEM; **p*<0.05, ***p*<0.01; ns, *p*>0.05. SI-JUND, Stereotaxic injection JUND (PLV3-CMV-mCherry-*JUND*) and glycine.

**Table S1. Characteristics of the including transcriptomic datasets of ASD.**

| Dataset | ASD | Control | Total | Brian tissue | Platform | PMID | Address |
| --- | --- | --- | --- | --- | --- | --- | --- |
| GSE178205 ^[1]^ | 13 | 10 | 23 | Cortex | GPL24676 | 35168652 | https://ftp.ncbi.nlm.nih.gov/geo/series/GSE178nnn/GSE178205/suppl/GSE178205_RNAseq_Brain_raw_counts.csv.gz |
| GSE28475 ^[2]^ | 10 | 9 | 19 | brain | GPL6883 | 21906392 | ftp://ftp.ncbi.nlm.nih.gov/geo/series/GSE28nnn/GSE28475/suppl/GSE28475_RAW.tar |
| GSE28521 ^[3]^ | 16 | 16 | 32 | frontal cortex | GPL6883 | 21614001 | ftp://ftp.ncbi.nlm.nih.gov/geo/series/GSE28nnn/GSE28521/suppl/GSE28521_RAW.tar |
| GSE38322 ^[4]^ | 14 | 12 | 26 | cerebellar | GPL10558 | 22984548 | ftp://ftp.ncbi.nlm.nih.gov/geo/series/GSE38nnn/GSE38322/suppl/GSE38322_RAW.tar |
| Total | 53 | 47 | 100 |  |  |  |  |

**Table S2. List of antibodies used for immunostaing.**

| Antibody | Company | Catalog number | Host species | Concentration |
| --- | --- | --- | --- | --- |
| Pax6 | CST | #60433 | Rabbit | 1:50 |
| β3-Tubulin | CST | #5568 | Rabbit | 1:200 |
| S100β | Proteintech | 15146-1-AP | Rabbit | 1:50 |
| GFAP | CST | #3670 | Mouse | 1:400 |
| GFAP | Abcam | ab4674 | Chicken | 1:100 |
| Vimentin | Affinity | #AF7013 | Rabbit | 1:500 |
| STAT3 | Bioss | bs-1141R | Rabbit | 1:100 |
| NeuN | Proteintech | 66836-1-Ig | Mouse | 1:50 |
| BAX | Proteintech | 50599-2-Ig | Rabbit | 1:2000 |
| BCL2 | Proteintech | 12789-1-AP | Rabbit | 1:1000 |
| Cleaved-Caspase 3 | Affinity | #AF7022 | Rabbit | 1:500 |
| Caspase 3/P17/P19 | Proteintech | 19677-1-AP | Rabbit | 1:1000 |
| Beta Actin | Proteintech | 66009-1-Ig | Mouse | 1:20000 |
| Goat anti-Mouse IgG1, Alexa Fluor™ 488 | Invitrogen | A21121 | Goat | 1:400 |
| Donkey anti-Rabbit IgG (H+L), Alexa Fluor™ 568 | Invitrogen | A10042 | Donkey | 1:500 |
| Goat Anti-Chicken IgY H&L (Alexa Fluor® 405) | Abcam | ab175674 | Goat | 1:100 |
| Goat Anti-Rabbit IgG H&L (HRP) | Abcam | ab6721 | Goat | 1:2500 |
| Goat Anti-Mouse IgG (HRP) | Merck | AP181P | Goat | 1:2500 |

**Table S3. Primers used for analysis of transcript levels.**

| **Target** | **Forward Primer** | **Reverse Primer** |
| --- | --- | --- |
| *JUND* | 5′-TCATCATCCAGTCCAACGGG-3′ | 5′-TTCTGCTTGTGTAAATCCTCCAG-3′ |
| *HSPB1* | 5′-TGGACCCCACCCAAGTTTC-3′ | 5′-CGGCAGTCTCATCGGATTTT-3′ |
| *GJA1* | 5′-GGTGACTGGAGCGCCTTAG-3′ | 5′-GCGCACATGAGAGATTGGGA-3′ |
| *BCL2* | 5′-GGTGGGGTCATGTGTGTGG-3′ | 5′CGGTTCAGGTACTCAGTCATCC-3′ |
| *BCL2L1* | 5′-GAGCTGGTGGTTGACTTTCTC-3′ | 5′-TCCATCTCCGATTCAGTCCCT-3′ |
| *FAS* | 5′-TCTGGTTCTTACGTCTGTTGC-3′ | 5′-CTGTGCAGTCCCTAGCTTTCC-3′ |
| *FOS* | 5′-CACTCCAAGCGGAGACAGAC-3′ | 5′-AGGTCATCAGGGATCTTGCAG-3′ |
| *PRNP* | 5′-AGTCAGTGGAACAAGCCGAG-3′ | 5′-CTGCCGAAATGTATGATGGGC-3′ |
| *EGR1* | 5′-AGTCCCATTTACTCAGCGGC-3′ | 5′-GTGGAAACAGGTAGTCGGGG-3′ |
| *APOE* | 5′-GTTGCTGGTCACATTCCTGG-3′ | 5′-GCAGGTAATCCCAAAAGCGAC-3′ |
| *IL-6* | 5′-CTGGGCAGACTCAAATTCCAGCTT-3′ | 5′-GCAAAGAGGCACTGGCAGAAAACA-3′ |
| *IL-17A* | 5′-GACAAGAACTTCCCCCGGAC-3′ | 5′-GCACTTTGCCTCCCAGATCA-3′ |
| *SLC1A2* | 5′-CCTGACGGTGTTTGGTGTCAT-3 | 5′-CAAGCGGCCACTAGCCTTAG-3′ |
| *GAPDH* | 5′-GACCTGACCTGCCGTCTA-3′ | 5′-AGGAGTGGGTGTCGCTGT-3′ |

**References**

1 Zhu Y, Gomez JA, Laufer BI, Mordaunt CE, Mouat JS, Soto DC, et al. Placental methylome reveals a 22q13.33 brain regulatory gene locus associated with autism. Genome Biol. 2022;23(1):46.

2 Chow ML, Li HR, Winn ME, April C, Barnes CC, Wynshaw-Boris A, et al. Genome-wide expression assay comparison across frozen and fixed postmortem brain tissue samples. BMC Genomics. 2011;12:449.

3 Voineagu I, Wang X, Johnston P, Lowe JK, Tian Y, Horvath S, et al. Transcriptomic analysis of autistic brain reveals convergent molecular pathology. Nature. 2011;474(7351):380-4.

4 Ginsberg MR, Rubin RA, Falcone T, Ting AH, Natowicz MR. Brain transcriptional and epigenetic associations with autism. PLoS One. 2012;7(9):e44736.
